# AZP2006 in Parkinson’s disease models: A promising new neuroprotective treatment involving lysosomal homeostasis and the Progranulin/Prosaposin complex

**DOI:** 10.1371/journal.pone.0359269

**Published:** 2026-09-30

**Authors:** Noelle Callizot, Georgia Culley, Grace Flower, Laura Rouvière, Véronique De Conto, Alexandre Wojcinski, Catherine Botto, Cecilia Estrella, Alexandre Henriques, Philippe Verwaerde

**Affiliations:** 1 Neuro-Sys, Chemin Départemental, Gardanne, France; 2 Alzprotect, Parc Eurasanté, Loos, France; University of Tartu, ESTONIA

## Abstract

The pathohistological hallmarks of Parkinson’s disease are aggregated alpha-synuclein and other aggregation-prone proteins (Lewy bodies). Recent studies show mechanistic as well as genetic connections between lysosomal dysfunction and Parkinson’s disease pathology. A direct link between lysosomal function and Parkinson’s disease might be the degradation of alpha-synuclein within the lysosomal system. Progranulin is necessary for maintaining lysosomal function, facilitating the activity of several lysosomal enzymes. Progranulin’s exit from the endoplasmic reticulum and its lysosomal availability depend on an interaction with Prosaposin. AZP2006 (INN: Ezeprogind) is a small lysosomotropic neuroprotective molecule currently in clinical development in Progressive Supranuclear Palsy patients. Its neuroprotective effects involve the Progranulin/Prosaposin complex. In this study, we investigated the neuroprotective effects of AZP2006 in several Parkinson’s-like models (*vitro* and *vivo*). We showed that AZP2006 was able to counteract a mitochondrial injury as well as the toxicity of alpha-synuclein preformed fibril spreading. We proved that Progranulin was involved in AZP2006’s neuroprotective action, restoring lysosomal homeostasis and ultimately supporting the health of dopaminergic neurons. In light of this evidence, strategies with the aim of improving Progranulin and Prosaposin levels and activity offer a promising therapeutic approach in the context of proteinopathies such as Parkinson’s disease.

## Introduction

Parkinson’s disease (PD) is defined by the progressive, irreversible loss of dopaminergic neurons in the substantia nigra pars compacta (*SN*pc). Its neuropathological hallmarks include the accumulation of misfolded, aggregated alpha-synuclein (α-syn) and other aggregation-prone proteins (Lewy bodies). Neurodegeneration in PD stems from multiple, interconnected mechanisms, such as proteasomal dysfunction-driven protein aggregation, mitochondrial stress, and impaired dopamine (DA) release. These pathways often converge, amplifying the production of reactive oxygen species (ROS) and oxidative stress, resulting in neuronal death [1].

More recent studies show mechanistic, as well as genetic, connections between lysosomal dysfunction and PD [2]. Lysosomes are acidic, membrane-enclosed organelles that degrade cellular macromolecules and participate in key processes such as endocytosis, phagocytosis and autophagy. Large protein aggregates, including α-syn, are predominately degraded via the lysosomal-autophagy system, where proteases, namely cathepsins, mediate their breakdown [3].

Lysosomal function depends on tightly regulated homeostasis mediated by lysosomal ion channels, which maintain the acidic pH required for lysosomal enzyme activity [4,5]. Progranulin (PGRN), is a ubiquitous growth factor which plays a role in regulating lysosomal function by enhancing the activity of lysosomal enzymes, including a critical protease, cathepsin D [6,7]. In the central nervous system, PGRN is highly expressed in microglia where it can be secreted as a full-length glycoprotein [8,9]. Once released, PGRN can interact with several signaling receptors [10] before becoming internalized and trafficked to the lysosomes of neurons and other glia [11]. PGRN deficiency disrupts microglial function and promotes neurodegeneration [12], whereas increasing PGRN levels in animal models of neurodegeneration enhances neuronal survival [13,14], axonal growth and regeneration after injury, and reduces oxidative stress [15]. Emerging clinical evidence links PGRN deficiency to neurodegenerative diseases whereby reduced PGRN levels have been reported in frontal temporal dementia (FTD), Alzheimer’s disease and PD [19–21].

The delivery of PGRN to the lysosome is dependent on the interaction with sortilin or prosaposin (PSAP) [11,16]. PSAP, a precursor of saposins (cofactors for glycosphingolipids hydrolases), is itself a lysosomal regulatory protein with neuroprotective properties [17,18]. In FTD caused by granulin (GRN) mutations [22], neuronal PSAP levels are diminished [23,24] — a finding that has been recapitulated in GRN-deficient mice [24]. Furthermore, PSAP supplementation has been shown to rescue lysosomal dysfunction in PGRN-deficient neurons [25], while PSAP-deficient mice exhibit reduced neuronal PGRN levels [25]. In PD, plasma PSAP levels decline with disease progression [21], further supporting the clinical relevance of this lysosomal protein. Collectively, these findings suggest that the PGRN-PSAP interaction is critical for lysosomal function and clearance of aggregation-prone proteins, such as α-syn.

The PGRN/PSAP complex is therefore a compelling target in PD. AZP2006 (INN: Ezeprogind; N-3-(4-(3-(diisobutylamino)propyl)piperazin-1-yl)propyl)-1H-benzo[d]imidazol-2-amine di-sulphate salt) is a small molecule that was initially screened and selected for its ability to reduce beta-amyloid (Aβ) release, increase levels of non-amyloidogenic APP metabolites and ultimately protect against neuronal loss [26]. AZP2006 is able to bind to the PGRN/PSAP complex and its neuroprotective effects are lost in the absence of either PGRN or PSAP [26,27]. These findings suggest that its neuroprotective mechanism relies on restoring lysosomal function through its interaction with the PGRN/PSAP complex.

AZP2006 is in development as a therapy for tauopathies, including Progressive Supranuclear Palsy (PSP), where a Phase 2a trial (NCT04008355) demonstrated favorable clinical outcomes [27]. Pre-clinical studies have also explored its potential in Alzheimer’s disease and FTD [26]. Here, we extend these investigations to PD by evaluating the neuroprotective effects of AZP2006 in multiple PD-like models (cellular and animal models) which reproduce key pathological features including mitochondrial stress, α-syn preformed fibril (PFF) toxicity and α-syn accumulation in neurons [28]. Given the critical role of microglia in PGRN secretion and activity, as well as its role in neuroinflammation, we also examined the impact of AZP2006 on microglial activation. *In vitro*, AZP2006 mitigated both acute mitochondrial injury, and chronic α-syn PFF-induced toxicity, which was associated with reduced microglial activation. These findings were translational *in vivo*, where AZP2006 protected dopaminergic neurons and reduced α-syn accumulation in aged mice injured with α-syn PFF. Using lysosomal function markers and PGRN knock-out (KO) cell lines, we provide evidence that the neuroprotective effects of AZP2006 in these PD models are mechanistically linked to the restoration of lysosomal homeostasis via the PGRN/PSAP complex.

## Materials and methods

All experiments were carried out in accordance with the National Institutes of Health Guide for the Care and Use of Laboratory Animals and followed current European Union regulations (Directive 2010/63/EU). Animals were housed for acclimation for 1 week in Neuro-Sys facilities and were maintained in a reversed 12 h light/dark cycle. They were placed in ventilated cages and maintained in a room with controlled temperature (21–22 °C), hygrometry (40–60%) and with food and water *ad libitum*.

### Primary culture and treatment of mesencephalic neurons in presence or absence of microglial cells

Pregnant female rats of 15 days gestation (Wistar rats, Janvier Labs, Le Genest-Saint-Isle, France) or mice of 14 days gestation (Swiss mice, Janvier Labs France) were sacrificed using deep anesthesia with CO_2_ followed by cervical dislocation. Rat or mouse mesencephalic neurons were prepared and cultured as previously described (see [1,29]). Briefly, the embryonic midbrains were dissected under a stereo zoom binocular microscope and placed in ice-cold medium of Leibovitz (L15, Pan Biotech, Aidenbach, Germany) containing 2% Penicillin (10,000 U/mL) and Streptomycin (10 mg/mL) solution (PS, Pan Biotech) and 1% bovine serum albumin (BSA, Pan Biotech). The ventral portion of the mesencephalic flexure, a region of the developing brain rich in dopaminergic neurons, was isolated for the cell preparations.

Midbrains were dissociated by trypsinization for 20 minutes at 37 °C (0.05% trypsin and 0.02% EDTA, Pan Biotech). The reaction was stopped by the addition of Dulbecco’s modified Eagle medium (DMEM, Dutscher, Bernolsheim, France) containing 0.2 mg/mL DNase I grade II (Pan Biotech) and 10% fetal calf serum (FCS, Thermo Fisher Scientific, Cergy Pontoise, France). Cells were then mechanically dissociated by 3 passages through a 10 mL pipette. Cells were centrifuged at 180 x *g* for 10 minutes at +4 °C on a layer of BSA (3.5%) in L15 medium. The supernatant was discarded and the cell pellets were re-suspended in a defined culture medium consisting of Neurobasal medium (Thermo Fisher Scientific) supplemented with: 2% B27 (Invitrogen), 2 mM L-glutamine (Pan Biotech), 2% PS solution, 10 ng/mL of Brain-derived neurotrophic factor (BDNF, Pan Biotech) and 1 ng/mL of Glial-Derived Neurotrophic Factor (GDNF, Pan Biotech). For the protocol using microglial cells, the medium was additionally supplemented with 4% heat-inactivated FCS, 1 g/L of glucose, 1 mM sodium pyruvate, and 100 µM of non-essential amino acids. Viable cells were counted using the LUNA-FL (logosbio) cell counter. The cells were seeded at a density of 40,000 cells/well (without microglial cells) or 80,000 cells/well (with microglial cells) in 96-well plates pre-coated with poly-L-lysine (Corning Biocoat, Le Pont de Claix, France) and maintained in a 5% CO_2_/95% air atmosphere humidified incubator at 37 °C. Half of the medium was replaced every 2 days with fresh medium.

On day 6, rat cultures were injured with 20 µM 6-Hydroxydopamine hydrobromide (6OHDA), 4 μM 1-Methyl-4-phenylpyridinium iodide (MPP^+^) or 10 nM Rotenone (all from Sigma-Aldrich, St. Louis, MO, USA), diluted in culture medium, in the presence of AZP2006 for 48 h (6OHDA and MPP^+^) or for 24 h (Rotenone). Mouse cultures were treated with an anti-PGRN antibody (5 μg/mL, ref: MAB2557) or an unrelated control for antibody treatment (anti-CD206, 5 μg/mL, ref: MCA2235GA (See S3 Fig)). For the study of lysosome exocytosis, cells were treated with 100 nM AZP2006 for 96 h (total), with treatment renewal after the first 48 h (day 8 of culture) and the culture stopped on day 10.

### Generation of PGRN-KO SH-SY5Y cell line and treatment

SH-SY5Y human neuroblastoma cells (Sigma Aldrich, 94030304) were cultured at Neuro-Sys (Gardanne, France). After a cell bank was generated, the cells were used between the 5^th^ and the 15^th^ passage. The PGRN knock-out (KO) SH-SY5Y cell line was generated by Neuro-Sys using CRISPR-Cas9 and homology directed repair approach. Briefly, CRISPR-Cas9 technology, using a non-homologous end joining approach (NHEJ), was used to generate an SH-SY5Y KO for GRN, the gene coding for PGRN protein. A single clone, selected based on the silencing of GRN gene, was characterized.

SH-SY5Y cells were transfected with a plasmid coding for single-guide RNA (sgRNA), single-stranded DNA (ssDNA) and a puromycin resistance gene. Transfected cells were selected with puromycin and grown from single cells into colonies. A bank of PGRN KO cells was then generated to allow future studies.

Cryoprotected wild-type (WT) and PGRN KO SH-SY5Y cells, stored in liquid nitrogen, were rapidly thawed in a water bath at 37 °C. Cells were immediately resuspended in high glucose-DMEM, (4.5 g/L, Dutscher), centrifuged at 125 x g for 7 minutes at 4 °C and the pellets resuspended in DMEM, supplemented with 10% FCS, and with 2% PS solution. Cells were seeded in 96-well plates at a density of 4,000 cells per well and cultured in a 5% CO_2_/95% air atmosphere humidified incubator at 37 °C. Cells were then cultured in DMEM, supplemented with 5% FCS and 1% PS solution. 10 μM retinoic acid (Sigma Aldrich) was added to the culture medium for 7 days to trigger cell differentiation. The culture medium was renewed every 2 days.

After one week of differentiation, WT SH-SY5Y cells were injured with 1 mM MPP^+^, whereas PGRN KO cells were injured with 500 μM MPP^+^, both for 48 h. Concentrations of MPP^+^ were chosen to achieve the same level of neuronal survival between WT and PGRN KO cell cultures after intoxication (S5 Fig). AZP2006 was administered in co-treatment.

### α-syn PFF preparation, injury and treatment of primary mesencephalic neurons (± microglia) or WT SH-SY5Y cells

Rat primary mesencephalic neurons + /- microglia or SH-SY5Y cells were prepared as previously described. Three days before injury, α-syn PFF were prepared following the procedure described by Henriques et al, 2022 [17]. Briefly, human α-syn peptide (stock at 69 µM in water at −20 °C) was reconstituted at a final concentration of 50 μM in NaCl 0.9% and gently agitated for 3 days at 37 °C in the dark to generate a preparation containing; α-syn monomeric form (27%), small oligomers (from 25–42 kDa; 30%) large oligomers/PFF (50–120 kDa; 30%) and large fibrils (14%), precisely quantified as previously described [17].

On day 6 (primary culture) or day 11 (SH-SY5Y cells) were intoxicated with the α-syn preparation (250 nM), in the presence of AZP2006, dissolved in culture medium, for 48 h (with microglia) or 96 h (without microglia) for primary culture and 72 h for WT SH-SY5Y cells. Following intoxication, the cell culture medium was removed, and cells were prepared for immunostaining.

### Immunocytochemistry and automated image analysis

#### Live cell imaging.

To identify functional lysosomes, we incubated WT SH-SY5Y cells with PhenoVue 577 (Revvity, diluted in Hank’s Balanced Salt Solution (HBSS)), which is a fluorescent dye that accumulates in acidic vesicles. An increase in fluorescence intensity is associated with increased lysosomal function. After 72 h of intoxication with α-syn PFF, culture medium was removed from WT SH-SY5Y cells and incubated with PhenoVue 577 for 30 minutes at 37 °C. HBSS was removed and images were acquired (see below for further details).

#### Fixed cell immunostaining.

At the end of the treatment, primary mesencephalic neurons cells were washed with phosphate-buffered saline (PBS, Dutscher) and fixed with 4% paraformaldehyde (PFA) in PBS (pH = 7.3), for 20 minutes at room temperature (RT). Cells were washed twice in PBS and then permeabilized and non-specific binding sites blocked with PBS containing 0.1% saponin and 1% FCS, for 15 minutes at RT. Cells were incubated with the following primary antibodies (Ab), in PBS containing 1% FCS, 0.1 mg/mL of saponin, for 2 h at RT: mouse monoclonal anti-Tyrosine Hydroxylase (TH, 1/10000) antibody or rabbit polyclonal anti-TH (1/2000) antibody to identify dopaminergic neurons and their neurites; polyclonal anti-α-syn antibody produced in rabbit or in guinea pig (1/200); polyclonal anti-post-synaptic density 95 (PSD95, a post-synaptic marker) produced in rabbit (1/400), polyclonal anti-lysosomal associated membrane protein 2 (LAMP2, marker of lysosomes) produced in rabbit (1/200) or in mouse; rabbit monoclonal anti-OX-41 antibody (to specifically stain microglial cells to study their activation (M1 phenotype)) (1/500) or with a monoclonal anti-light chain 3 b (LC3b) antibody produced in mouse (1/100), a marker of autophagy (see Table 1 for more details).

**Table 1:** Antibodies used for *in vitro* investigations.

Antibody	Species	Mono/Poly	Manufacturer	Catalogue no.	RRID	Figure
TH	Mouse	Monoclonal	Sigma-Aldrich	T1299	AB_477560	1, 2, 3, 4
TH	Rabbit	Polyclonal	Sigma-Aldrich	AB152	AB_390204	1
TH	Chicken	Polyclonal	Abcam	Ab76442	AB_1524535	3, 7
PGRN	Mouse	Monoclonal	Biotechne	MAB2557	AB_3657952	1
α-syn	Rabbit	Polyclonal	Ozyme	2642S	-	1, 2, 6
α-syn	Guinea pig	Polyclonal	Alomone	APZ-035-GP	AB_2925147	3
LC3b	Mouse	Monoclonal	Ozyme	83506S	AB_2800018	3
PSD95	Rabbit	Polyclonal	Ozyme	2507S	–	1
LAMP2	Rabbit	Polyclonal	Abcam	Ab203224	–	2, 3, 7
LAMP2	Mouse	Monoclonal	Fisher Scientific	15812694	–	6
OX-41	Rabbit	Monoclonal	Novus	NBP1–03074	–	4
CD206	Rat	Monoclonal	Bio-rad	MCA2235GA	AB_322613	S3 Fig

For WT and PGRN-KO SH-SY5Y cells injured with MPP^+^, after 48 h of intoxication, cells were fixed with methanol. After being washed twice in PBS, cell membrane permeabilization and blocking of non-specific binding sites was performed with a solution of PBS containing 5% BSA and 0.1% Triton X-100 for 10 minutes at RT. Cells were then incubated with the following primary antibodies: a polyclonal anti-α-syn antibody, produced in rabbit, at a dilution of 1/200 and mouse anti-LAMP2 antibody, at a dilution of 1/1000.

Following primary antibody incubation (primary mesencephalic neurons or SH-SY5Y cells), cells were washed with PBS containing 1% FCS, 0.1% saponin, and then incubated with the appropriate secondary antibodies: Alexa Fluor conjugated antibodies, diluted 1/400 in PBS containing 1% FCS, 0.1% saponin, for 1 hour at RT. Cell nuclei were counterstained with the fluorescent Hoechst dye (1/1000, Sigma Aldrich).

#### Automated image analysis.

For each experimental condition (6 culture well replicates), images representing 80% of the total surface of the well area were automatically acquired at 10x magnification with ImageXpress® (20 photomicrographs per well, Molecular Devices, San Jose, CA, USA) or Operetta® (9 photomicrographs per well, Revvity), using the same acquisition parameters. Image analysis was performed automatically using MetaXpress® (Molecular Devices) or Harmony® (Revvity). Fluorescence detection thresholds were established based on background signal from control cultures treated only with secondary antibodies; since secondary antibodies did not produce detectable signal, this threshold was set at a low baseline level. Read-outs included: the number of TH(+) neurons (survival) and the length of their neurite network (µm); the area of cytoplasmic α-syn accumulation, the area of LAMP2(+) vesicles, the overlapping area between α-syn and LAMP2(+), LC3b(+) vesicles within the zone of interest, the overlapping area between LAMP2(+) vesicles and LC3b(+) vesicle area within TH(+) neurons (in µm²), PhenoVue 577(+) area (in µm²) within WT SH-SY5Y cells, as well as, the area of OX-41(+) staining in microglial cells. Further description of segmentation analysis can be found in the supplementary information (S1 Fig).

For the analysis of lysosomal cellular distribution, at least 4 representative images for each condition were acquired using a confocal microscope (LSM 900, Zeiss) with Zen software at 63x magnification, using identical acquisition parameters (automatic acquisition; pixel resolution = 71 nm x 71 nm per pixel). Representative images were generated to evaluate the overlap between TH and LAMP2, at the cell membrane. The total number of LAMP2(+) lysosomes, and their subcellular localization (membrane or cytoplasm), in TH(+) neurons was analyzed using ImageJ software.

Results are expressed in terms of the percentage relative to control conditions (i.e., number of neurons). Each experiment consists of one culture (3 immunocytochemistry (ICC) markers + Hoechst stain per culture) and numbers are reported as biological replicates (n = 4–6 / group).

### Intranigral injection of α-syn preparation in mice and treatments

C57BL/6 male mice (18-month-old) were provided by Charles River Laboratory (Saint Germain Nuelles, France) and acclimated for one-week. Anesthesia was induced with 4% isoflurane (CentraVet, Lapalisse, France) in an induction chamber coupled with a vaporizer and an oxygen concentrator. Mice were then placed in the stereotaxic frame and anesthesia was maintained by 2% isoflurane delivered via a face mask. After the skull was exposed and holes were drilled, bilateral stereotaxic injections of the α-syn preparation reconstituted as previously described for *in vitro* experiments, 2.5 µL/injection site, 50 µM) or vehicle (NaCl 0.9%) were performed with a Hamilton syringe (0.1 µL/minute with an Elite Nanomite syringe pump). Injections targeted the SNpc at the following coordinates: A-P: −3 mm; M-L: ± 1.2 mm; D-V:-4.5 mm according to the Paxinos and Franklin’s mouse brain atlas [30]. The SNpc was targeted to allow direct contact between α-syn oligomers/fibrils and the dopaminergic neurons’ cell bodies. Further details of the model are described in Henriques et al., 2022 [28].

Depth of anesthesia and rectal temperature were verified every 5 minutes throughout the procedure. After surgery, mice were allowed to recover before being placed back in their cage.

An initial study was performed to characterize the effects of α-syn infusion into the SNpc of aged mice and determine the effect size necessary for the given study. Vehicle-infused and α-syn-infused mice were sacrificed at 2, 4 or 5 weeks after surgery (n = 7 mice /group, S5 Fig). The results obtained on TH(+) neurons survival, α-syn accumulation in these neurons and Iba1(+) microglia activation, informed the decision to choose week 5 (W5) as the optimal timepoint for evaluating the efficacy of AZP2006.

Subsequently, AZP2006 (2 mg/kg; dose selected according to previous studies, Callizot et al., 2021) or vehicle (deionized water) were administered *per os* (p.o.) by gavage once daily, from the day of the surgery, for 5 weeks until the end of the study. Experimental groups include: sham/vehicle, α-syn/vehicle and α-syn/AZP2006 (2 mg/kg/d); n = 7 mice /group.

Based on previous work in this model [28], power analysis (effect size = 2; alpha = 0.05; power = 0.9) indicated that for histological analysis, n = 7 mice / group are necessary to detect a statistical difference between two groups.

### Tissue collection and immunohistochemistry (IHC)

At 5 weeks post-surgery, 2 h after the final administration of AZP2006 (Fig 5), mice were deeply anesthetized and perfused with cold PBS (3 minutes), followed by cold PFA 4% in PBS (3 minutes) (for results at 2, 4 or 5 weeks after surgery, see S1 Fig). Brains were dissected and post-fixed in PFA 4%, overnight at 4 °C, then cryoprotected in 30% sucrose in PBS at 4 °C. Coronal sections, including the SNpc, were cut using a cryostat at either 40 µm-thickness (4 sections per mouse, each 100 µm apart, for α-syn injury characterization) or at 20 μm-thickness (6 sections per mouse, each 160 μm apart, for AZP2006 treatment analysis). Free-floating sections were incubated in PBS with 0.25% BSA, 0.3% Triton X-100 and 1% goat serum, for 1 hour at RT, to block unspecific binding sites and permeabilize the tissue.

Sections were incubated for 24 h or overnight at 4 °C, or for 2 h at RT, with selected primary antibodies (See Table 2). Briefly, brain sections were stained with: chicken polyclonal anti-TH antibody (1/1000, Abcam), rabbit polyclonal anti-α-syn antibody (1/200, Cell Signaling), rat monoclonal anti-ionized calcium binding adapter molecule 1 (Iba1) antibody (1/200, Abcam) or rabbit polyclonal anti-Iba1 antibody (1/500, Novus). These antibodies were revealed with: Alexa Fluor 488 anti-rabbit IFgG, Alexa Fluor 568 anti-chicken IgY, or Alexa Fluor 647 anti-goat IgG at the dilution 1/500 (all from Sigma) in PBS or tris buffered saline (TBS) with 0.25% donkey serum or BSA, 0.3% Triton X-100 and 1% goat serum.

**Table 2:** Antibodies used for *in vivo* investigations.

Antibody	Species	Mono/Poly	Manufacturer	Catalogue no.	RRID	Figure
TH	Chicken	Polyclonal	Abcam	Ab76442	AB_1524535	5, S4 Fig
α-syn	Rabbit	Polyclonal	Cell Signaling	2642S	-	5, S4 Fig
Iba1	Rat	Monoclonal	Abcam	Ab283346	AB_3065282	5
Iba1	Rabbit	Polyclonal	Novus	NBP2–19019	AB_3073939	S4 Fig

Images were automatically acquired with a confocal microscope (LSM 900) with Zen software at 20x magnification, using the same acquisition parameters across all samples. Image analysis was automatically performed using MetaXpress®. The following read-outs were measured in the SNpc: the number of TH(+) neurons (survival), the accumulation of α-syn in TH(+) cells and the number of Iba1(+) microglial cells.

An overview of all the models used to assess the effects of AZP2006 can be found in the supplementary information (S2 Fig).

### Statistical analysis

Data are expressed as mean ± SEM (standard error of the mean). Shapiro-Wilks normality test was run for each data set. As the data were normally distributed, statistical analysis was performed by a one-way ANOVA or two-way ANOVA, followed by a Fisher’s LSD test, or Student *t*-test, as appropriate, using GraphPad Prism (version 10).

## Results

### Neuroprotective effects of AZP2006 on TH(+) neurons in Dopaminergic-toxin models

Oxidative stress-mediated apoptotic cell death is a major contributing factor to the selective degeneration of an early oxidative stress and activation of caspase 3, leading to apoptotic cell death within the 24 h. In addition, α-syn aggregates accumulate in the cytoplasm of TH(+) neurons after toxin exposure [1]. AZP2006 added concomitantly with 6OHDA was able to prevent neuronal cell death concentration-dependently, with a maximal effect reached at 100 nM. Similarly, AZP2006 fully protected the neurite integrity, as well as protecting synapses (indicated by PSD95 staining). AZP2006 completely abolished α-syn accumulation induced by 6-OHDA (Fig 1A-1D). Interestingly, in the presence of an anti-PGRN Ab (added in excess in the culture medium), the neuroprotective effect was highly decreased (Fig 1E-1H). To ensure the effects of anti-PGRN Ab were specific, we applied an Ab that was unrelated to PGRN, to any cellular or subcellular targets within the culture or the proposed mechanism of AZP2006 (anti-CD206 antibody) and found the neuroprotective effects of AZP2006 remained unchanged (S3 Fig).

**Fig 1:**
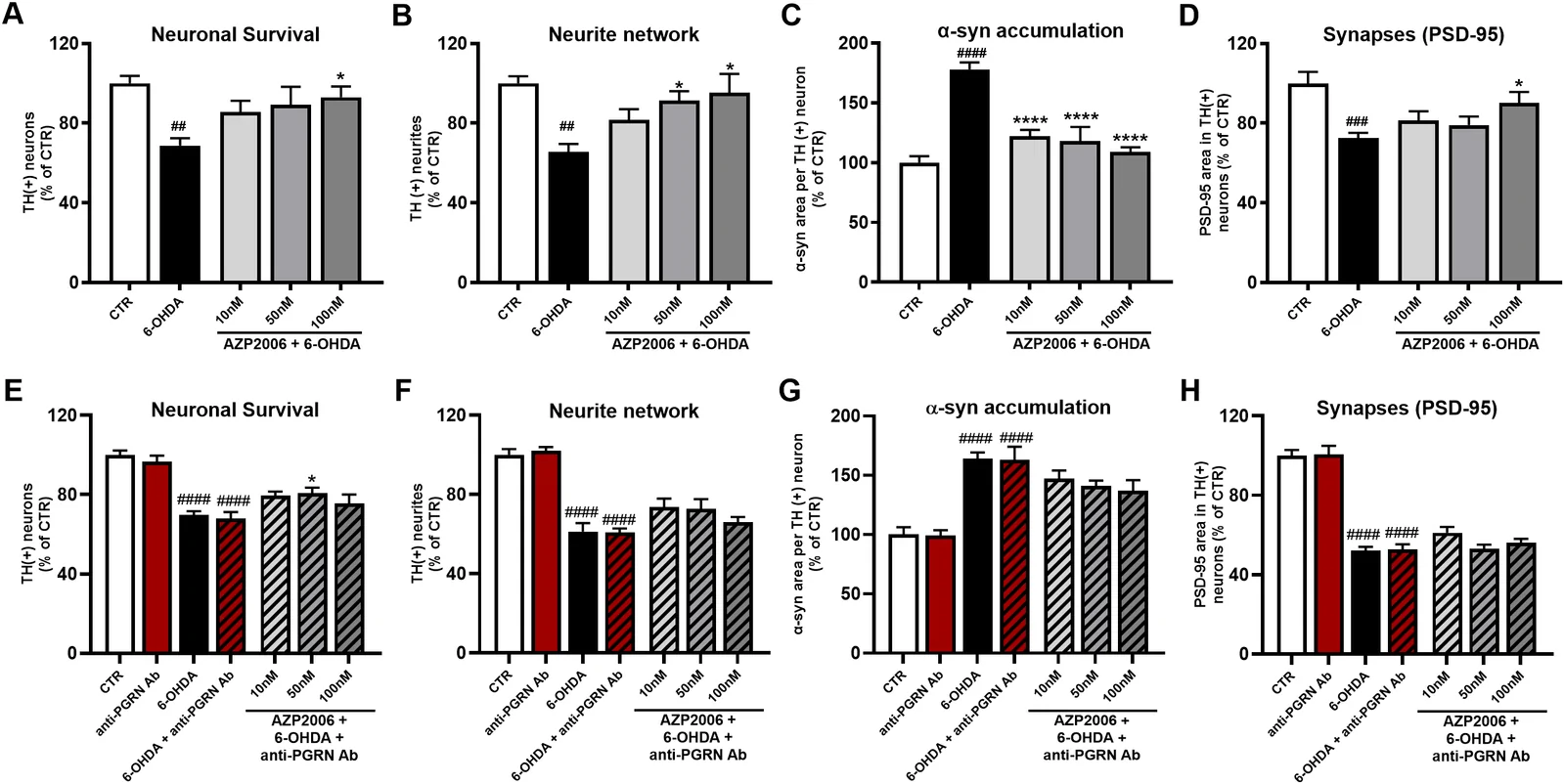
Effects of AZP2006 on TH(+) primary mesencephalic neurons injured with 6OHDA (20 µM) for 48 h, in the absence (A-D) or presence (E-H) of anti-PGRN Ab. ICC analysis of cultures shows the total number of TH(+) neurons (A,E), the length of their neurite network (B,F), the area of cytoplasmic α-syn staining per TH(+) neuron (C,G) and the area of the post-synaptic marker PSD95 per TH(+) neuron (D and H), expressed as % of control (CTR). Raw values for CTR (i.e., 100%); sum TH(+) neurons per well, i.e., per biological replicate = 27 ± 1.0; neurite network = 3540 ± 127 µm; α-syn area/TH(+) neuron = 201 ± 8.9 µm^2^; PSD95 area/TH(+) neuron = 1829 ± 105 µm^2^. Each bar represents the mean ± SEM, one-way ANOVA followed by Fisher’s test. ^##^p < 0.01; ^###^p < 0.001 or ^####^p < 0.0001 from CTR (control) or from anti-PGRN Ab and *p < 0.05 or ****p < 0.0001 from 6OHDA or from 6OHDA + anti-PGRN Ab; (n = 4-6 / group).

MPP^+^ (a mitochondrial complex I inhibitor) induces a moderate oxidative stress and stimulates caspase-independent cell death. AZP2006 was able to protect TH(+) neurons and their neurite network from MPP^+^ injury and also decreased α-syn accumulation. Interestingly, lysosomal stress (represented by the area per neuron of LAMP2 (+) vesicles, see Henriques et al. 2022) was highly reduced with AZP2006 (Fig 2A-2D).

**Fig 2:**
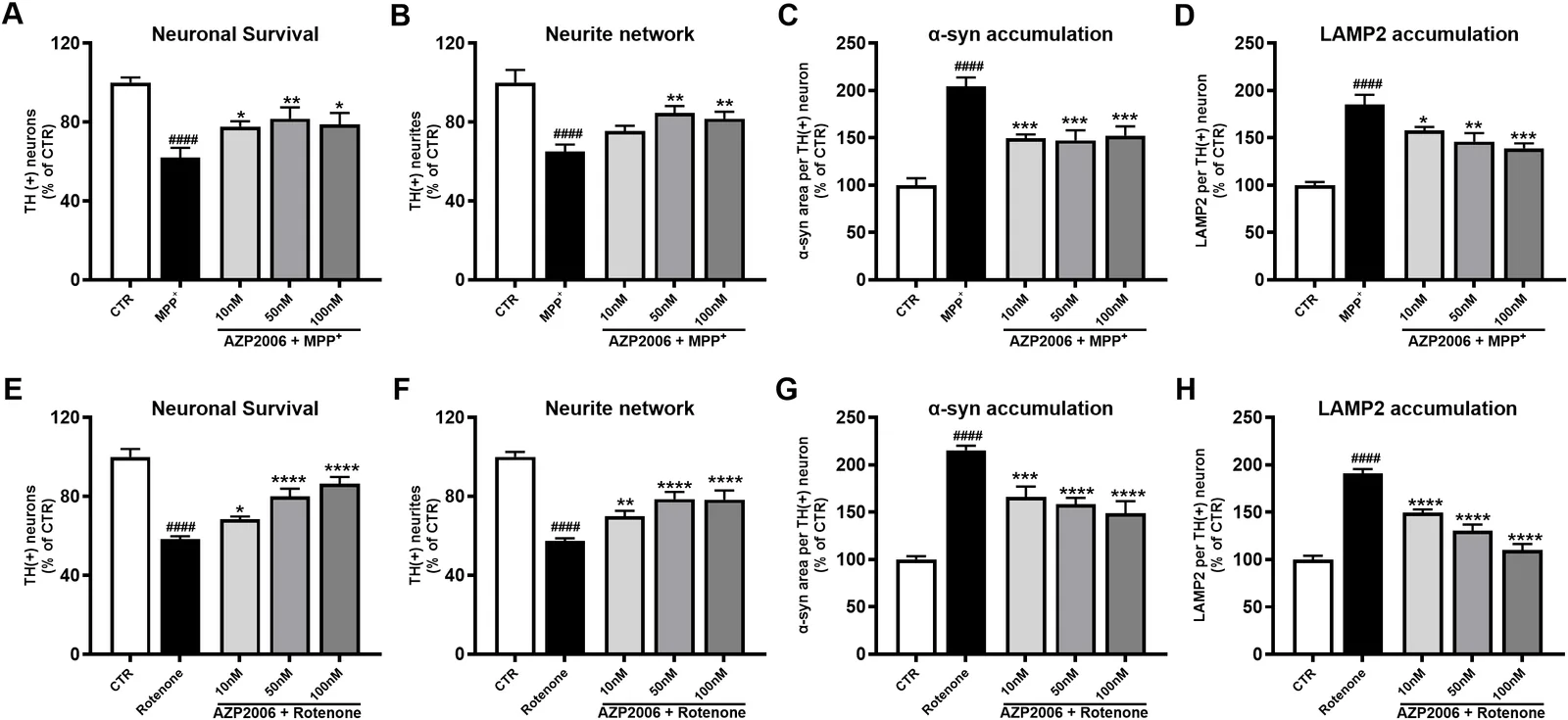
Effects of AZP2006 on TH(+) primary mesencephalic neurons injured by MPP^+^ (4 µM) for 48 h (A-D) or with Rotenone (10 nM) for 24 h (E-H). ICC analysis of cultures shows the total number of TH(+) neurons (A,E), the length of their neurite network (B,F), the area of cytoplasmic α-syn per TH(+) neuron (C,G) and lysosome burden as area of LAMP2(+) vesicles per TH(+) neuron (D and H), expressed as % of control (CTR). Raw values for MPP^+^ & Rotenone CTR (i.e., 100%); sum TH(+) neurons per well, i.e., per biological replicate = 18 ± 0.5 & 26 ± 1; neurite network = 889 ± 57 & 929 ± 24 µm; α-syn area/TH(+) neuron = 29 ± 2.1 & 28 ± 1 µm^2^; ratio LAMP2/TH(+) = 10 ± 0.3 & 10 ± 0.5, respectively. Each bar represents the mean ± SEM, one-way ANOVA followed by Fisher’s test. ^####^p < 0.0001 from CTR and *p < 0.05, **p < 0.01; ***p < 0.001 or ****p < 0.0001 from MPP^+^ or Rotenone treatment, (n = 4-6 / group).

Rotenone induces a substantial necrosis of neurons at high concentrations, whereas at low concentrations it only triggers apoptosis. It induces a depletion of ATP pools and a large cytochrome C release, soon after treatment [1]. AZP2006 protected TH(+) neurons from rotenone (10 nM) injury, preventing neurite network loss, α-syn accumulation, reducing the massive lysosomal stress of TH(+) neurons and finally preventing neuronal death (Fig 2E-2H).

### Neuroprotective effects of AZP2006 on TH(+) neurons after α-syn PFF exposure

α-syn PFF are known to induce progressive cytotoxicity, characterized by mitochondrial dysfunction and oxidative stress [28]. Concomitantly, an increase LAMP2, a marker of lysosomal vesicles, is observed, suggesting that exogenous α-syn travels to the lysosome via the endosome for clearance, as a mechanism to reduce α-syn levels. Interestingly, 96 h after the α-syn PFF application, a large lysosomal burden is observed, in conjunction with reduced lysosomal function (after 72 h), leading to extensive neuronal death [28].

AZP2006 significantly reduced the large accumulation of both lysosomes (LAMP2(+)) and autophagosomes (LC3b, evaluated by LC3b(+) vesicles), after a 96-h injury with the α-syn PFF preparation (Fig 3D and 3F). AZP2006 also attenuates the increase in autolysosomes (fusion of lysosomes with autophagosomes (overlapping LAMP2(+)/ LC3b(+) signal)) (Fig 3G). AZP2006 decreased the level of α-syn accumulated within lysosomes in a concentration-dependent manner (Fig 3E). These results are attributed to an increase in lysosomal function following AZP2006, in a concentration-dependent manner (Fig 3H).

**Fig 3:**
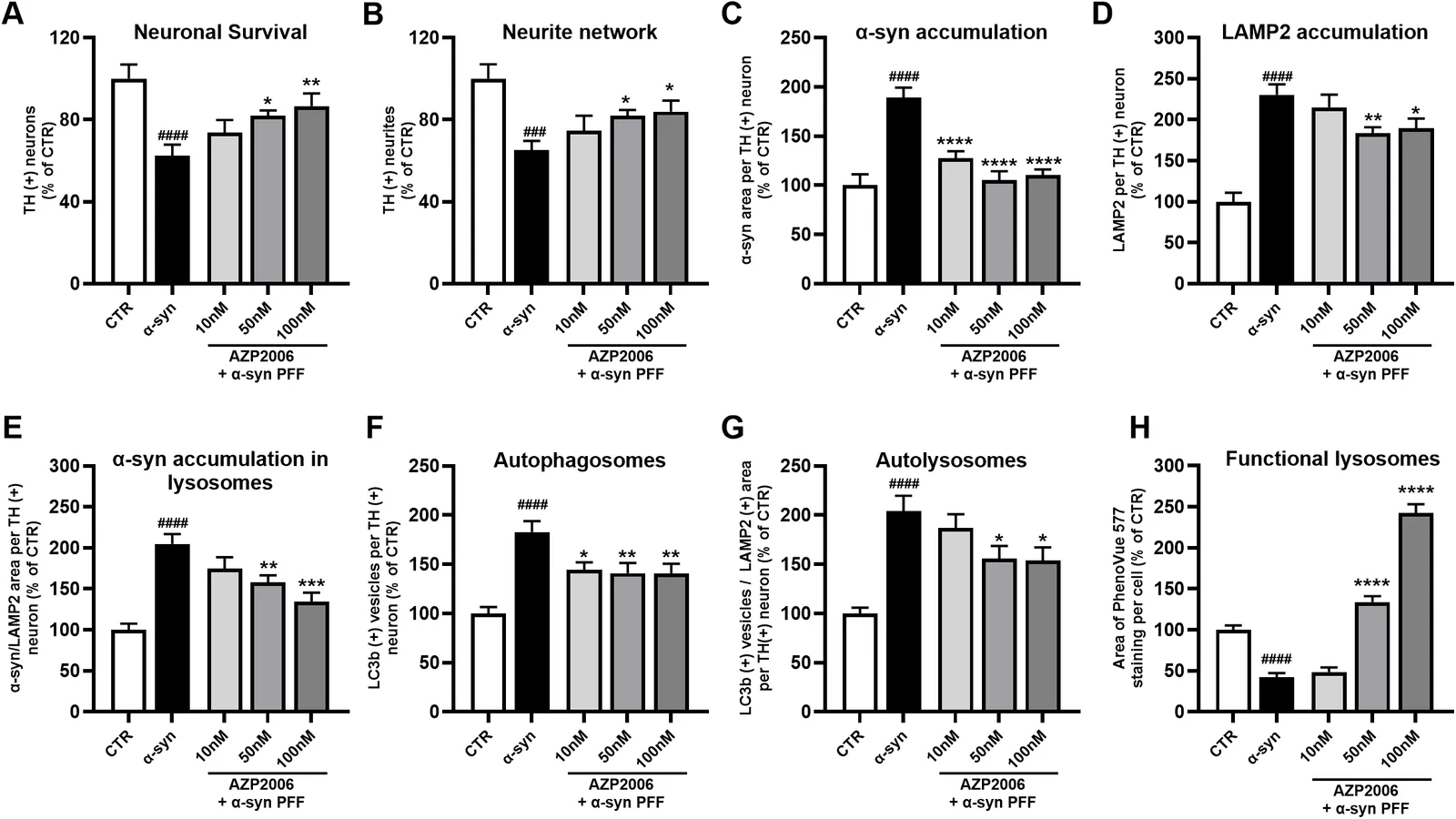
Effects of AZP2006 on TH(+) primary mesencephalic neurons cultured without microglia (A-G) or WT SH-SY5Y cells (H) and injured by α-syn PFF preparation for 96 h (primary cultures) or 72 h (SH-SY5Y cells). ICC analysis of cultures shows the total number of TH(+) neurons (A), the length of their neurite network (B), the area of cytoplasmic α-syn staining per neuron (C), the area of LAMP2(+) staining per TH(+) neuron (D), the α-syn accumulation in lysosomes as α-syn/LAMP2 colocalization area (E), autophagosomes as LC3b(+) vesicles per TH(+) neuron (F), autolysosomes as LC3b(+) vesicles colocalized with LAMP2 area in TH(+) neurons (G), and functional lysosomes expressed as area of PhenoVue 577(+) staining per SH-SY5Y cell (H), expressed as % of control (CTR). Raw values for CTR (i.e., 100%); sum TH(+) neurons per well, i.e., per biological replicate = 32 ± 1.3; neurite network = 1054 ± 60.6 µm; α-syn area/TH(+) neuron = 25.2 ± 2.8 µm^2^; LAMP2 area/TH(+) neurons = 17.9 ± 1.5 µm^2^; α-syn area/LAMP2/TH(+) neuron = 4.4 ± 0.3 µm^2^; LC3b area/TH(+) neurons = 27 ± 1.7 µm^2^; LAMP2/LC3b area/TH(+) neurons = 7.8 ± 0.5 µm^2^; PhenoVue 577(+) staining/SH-SY5Y cell = 67 ± 3.5 µm^2^. Each bar represents the mean ± SEM, one-way ANOVA followed by Fisher’s test. ^###^p < 0.001 or ^####^p < 0.0001 from CTR; *p < 0.05, **p < 0.01, ***p < 0.001 and ****p < 0.0001 from the α-syn condition, (n = 5-6).

In parallel experiments using co-cultures with microglial cells, α-syn PFF application for 48 h lead to a pronounced microglial activation associated with a large neuronal death. In the presence of AZP2006, inflammation was significantly reduced, in addition TH(+) neurons (number and their neurite network) were preserved from the α-syn-induced inflammation (Fig 4A-4C).

**Fig 4:**
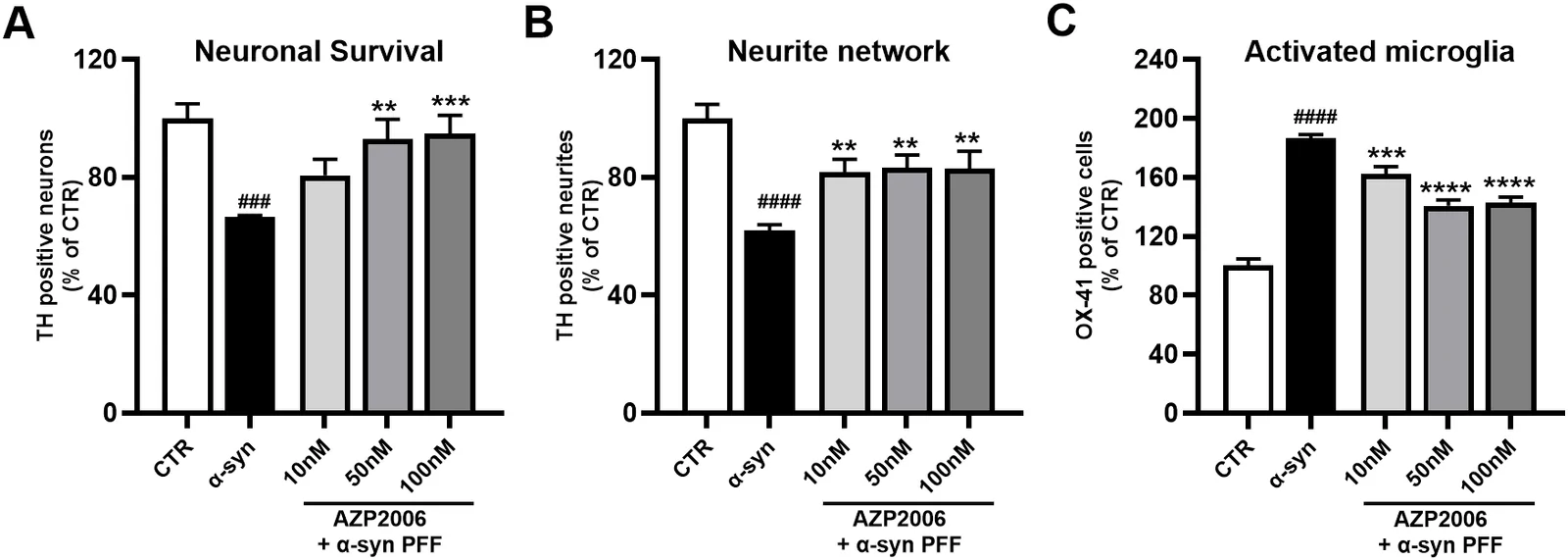
Effects of AZP2006 on TH(+) primary mesencephalic neurons cultured with microglia and injured by α-syn PFF preparation for 48 h. ICC analysis of cultures shows the total number of TH(+) neurons (A) and the length of their neurite network neurons (B) and microglia activation as area of OX-41(+) staining (C), expressed as % of control. Raw values for control (CTR) (i.e., 100%); sum TH(+) neurons per well, i.e., per biological replicate = 81 ± 4.0; neurite network = 2332 ± 112 µm; OX-41(+) area/field = 1421 ± 67 µm^2^. Each bar represents the mean ± SEM, one-way ANOVA followed by Fisher’s test. ^###^p < 0.001 or ^####^p < 0.0001 from CTR; **p < 0.01, ***p < 0.001 and ****p < 0.0001 from α-syn injury, (n = 4-6 / group).

### *In vivo* effects of AZP2006 on Aged mice injured with α-syn PFF

Bilateral injection of α-syn PFF preparation into the SNpc of aged animals induced progressive accumulation and spreading of α-syn, associated with loss of TH(+) neurons and microglial activation over a 5-week period (see supplementary results, S4 Fig), consistent with previous findings [17].

Daily administration of AZP2006 for 5 weeks post-lesion showed no effect on body mass (Fig 5A). After 5 weeks, AZP2006 significantly counteracted the TH(+) neuronal loss occurring in the SNpc (Fig 5C) and pathological α-syn accumulation was fully prevented in the cytoplasm of TH(+) neurons (Fig 5D). In addition, a complete inhibition of microglial activation was observed (Fig 5E). These findings are illustrated with representative images (Fig 5B) for control, injured and injured + AZP2006-treated conditions.

**Fig 5:**
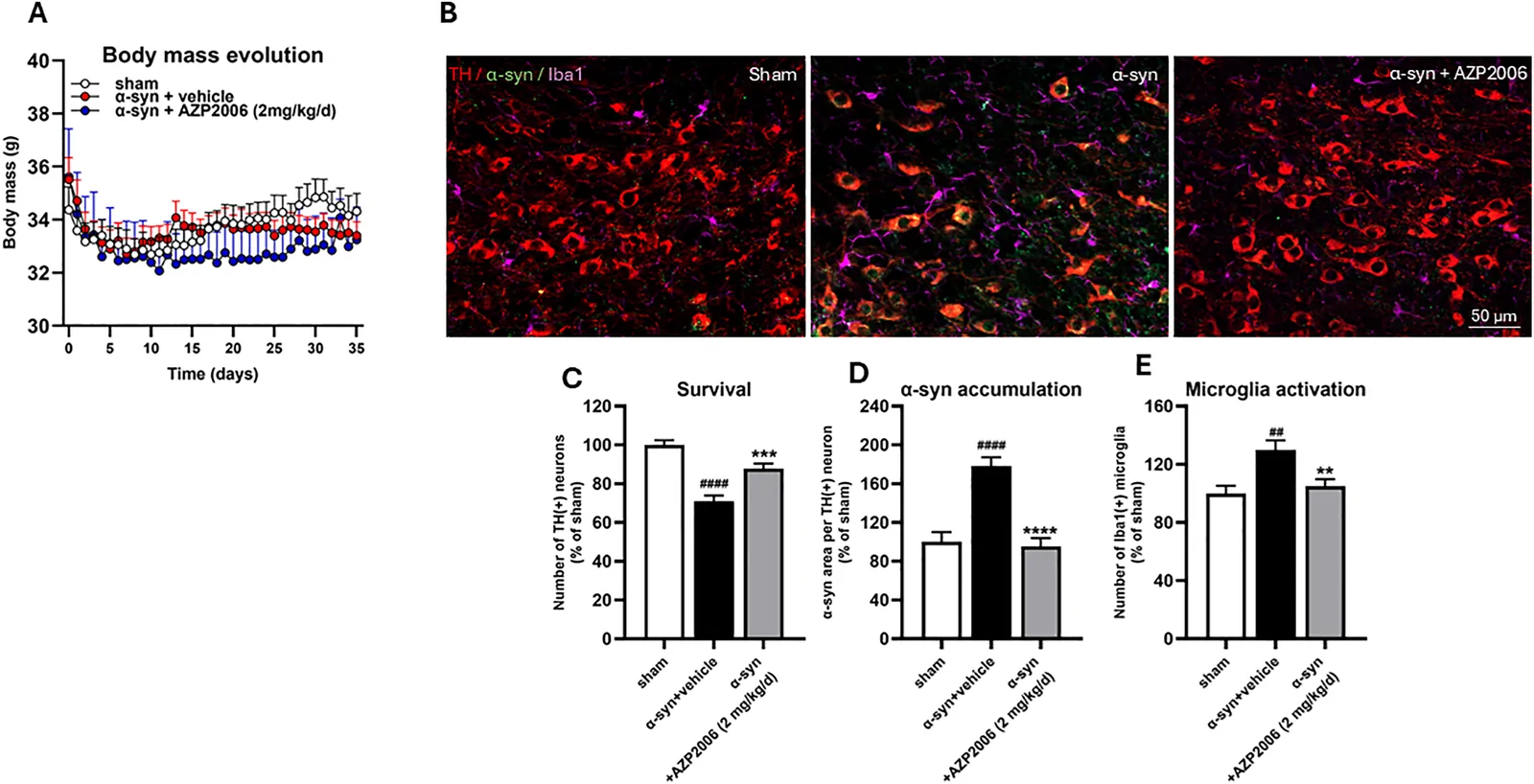
Effects of AZP2006 in aged mice infused with α-syn PFF preparation in the SNpc, treated for 5 weeks. Body mass evolution during 35 days of daily treatment with vehicle or AZP2006 post-lesion (A). Representative IHC images TH (red)/α-syn (green)/Iba1 (purple) (B). IHC analysis shows the total number of TH(+) neurons (C), area of α-syn accumulation in TH(+) neurons (D) and the number of Iba1(+) microglia in the SNpc (E) expressed as % of sham. Raw values for sham (i.e., 100%); TH(+) neurons = 65 ± 1.6; α-syn area/TH(+) neuron = 168 ± 9.4 µm^2^; Iba1(+) cells = 53 ± 2.8. Each bar represents the mean ± SEM, one-way ANOVA followed by Fisher’s test. ^##^p < 0.01 or ^####^p < 0.0001 from sham; **p < 0.01; ***p < 0.001 and ****p < 0.0001 from α-syn+vehicle injury, (n = 7 mice / group).

### Mechanistic study of AZP2006 on WT and PGRN-KO SH-SY5Y cells after MPP^+^ exposure

PGRN-KO SH-SY5Y cells were generated and subsequently differentiated into neuron-like cells by the application of retinoic acid for 7 days. PGRN-KO and WT cell cultures were subjected to a high concentration of MPP^+^ (500 µM and 1mM, respectively) for 48 h in the presence or absence of AZP2006, added at the same time as MPP^+^. Concentrations of MPP^+^ were chosen to achieve a similar level of neuronal survival between WT and PGRN KO cell cultures after intoxication (S5 Fig.).

AZP2006 (at all tested concentrations) prevented cell death and reduced α-syn accumulation in WT SH-SY5Y cells. Lysosomal accumulation was fully abolished in the presence of AZP2006 (Fig 6A-6C). In contrast, AZP2006 was unable to prevent cell death or α-syn accumulation in PGRN-KO SH-SY5Y cells (Fig 6D and 6E). In addition, the large lysosomal stress (LAMP2 accumulation) induced by MPP^+^ injury was not alleviated by AZP2006 at any concentration (Fig 6F). These results highlight the crucial role of PGRN in mediating the neuroprotective action of AZP2006: in the absence of PGRN, AZP2006 was unable to mitigate lysosomal stress or neuronal damage.

**Fig 6:**
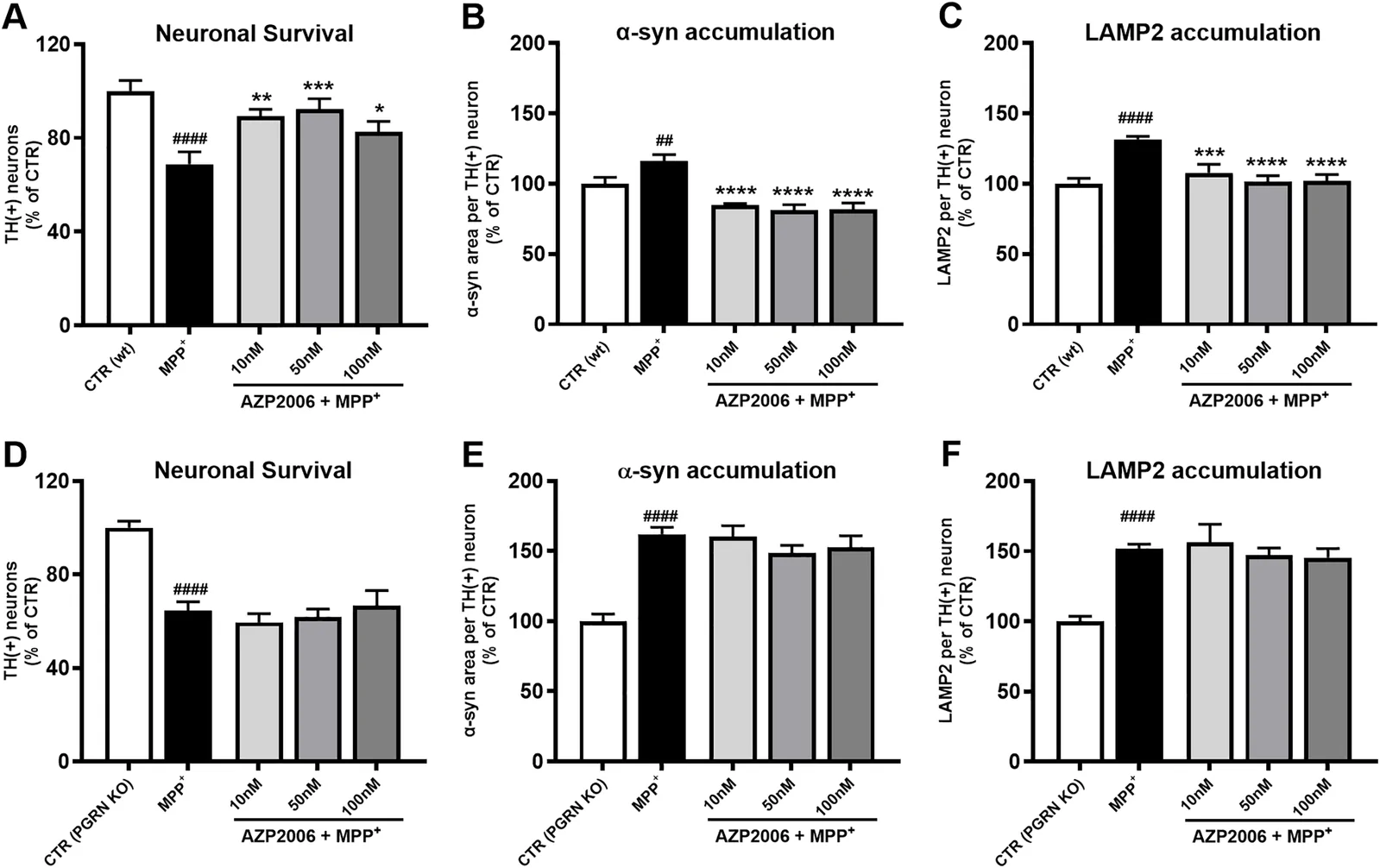
Effects of AZP2006 on WT and PGRN-KO SH-SY5Y cells injured by MPP^+^ 1 mM (A-C) and 500 µM (D-F) for 48 h. ICC analysis shows the total number of cells (A, D), the area of cytoplasmic α-syn staining per cell (B,E) and lysosomal burden as area of LAMP2(+) vesicles per cell (C,F), expressed as % of control (CTR = PGRN KO alone in D-F). Raw values for PGRN KO CTR (i.e., 100%); cells/image field = 140 ± 3.9; α-syn area/cell = 38.1 ± 2.8 µm^2^; LAMP2(+) area/cell = 51 ± 2.8 µm^2^. Each bar represents the mean ± SEM, one-way ANOVA followed by Fisher’s test. ^##^p < 0.01 or ^####^p < 0.0001 from CTR and *p < 0.05, **p < 0.01; ***p < 0.001 or ****p < 0.0001 from MPP^+^ (n = 4-6 / group).

In primary TH(+) neurons, incubation with AZP2006 at a neuroprotective concentration (100 nM, for 96 h), significantly increased LAMP2 levels within the membrane of neurons, whereas cytoplasmic lysosome levels were not significantly modified (Fig 7C and 7D). A non-significant increase of total lysosome number was observed (Fig 7B). These results support the lysosomal tropism of AZP2006 and its lysosomal accumulation (consistent with previous findings; 15), suggesting its role in promoting lysosomal fusion with the plasma membrane.

**Fig 7:**
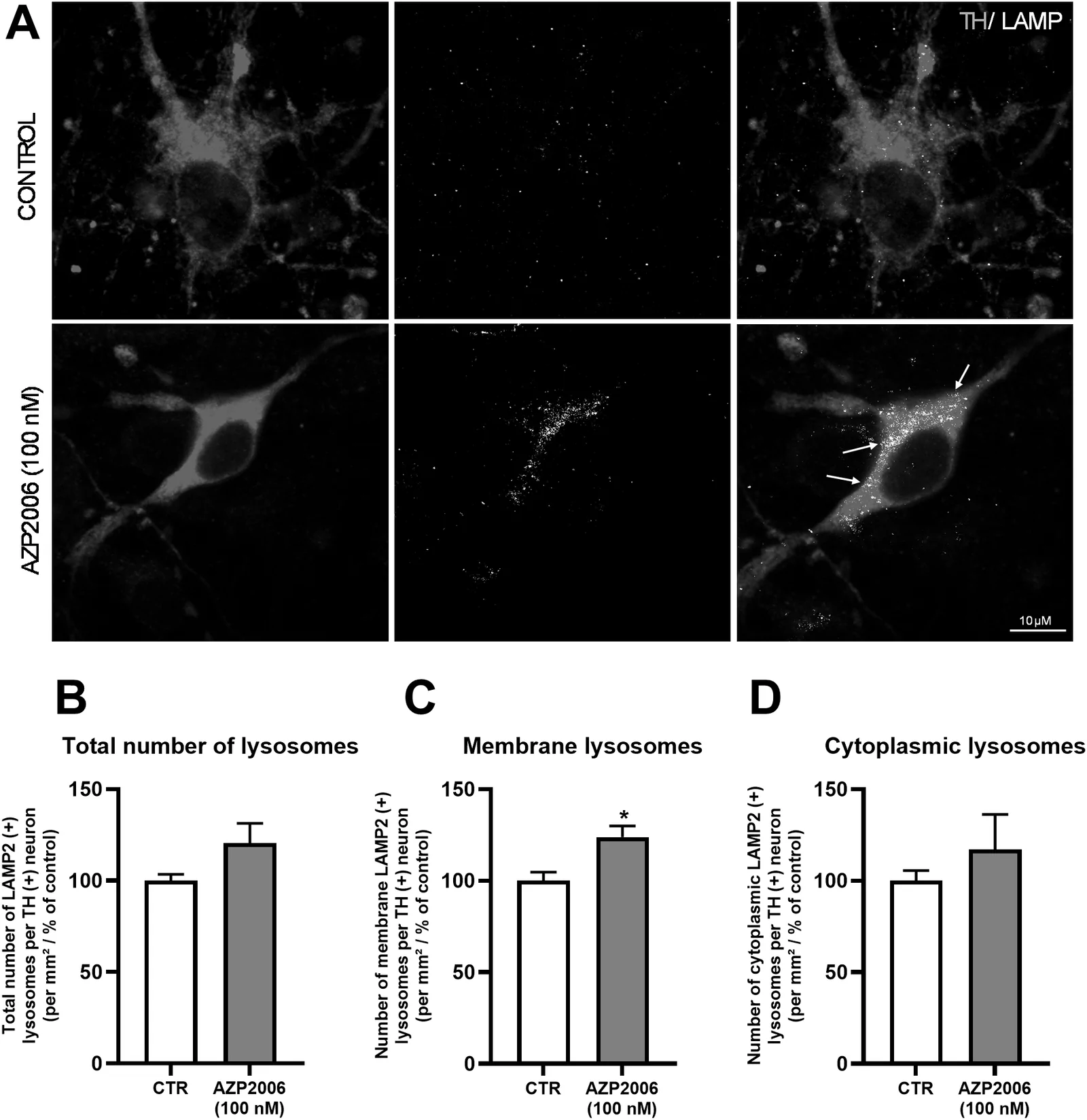
Effects of AZP2006 on lysosomal exocytosis in TH(+) primary mesencephalic neurons after a 96 h application. A) ICC representative images of LAMP2(+) vesicles (red) in TH(+) neurons (A) in CTR (control) and AZP2006 (100 nM) treated cells (63x magnification). ICC analysis shows the total number of lysosomes (B) per TH(+) neuron and the cellular distribution of the LAMP2(+) lysosomes at the membrane (C), and at the cytoplasm (D). Raw values for CTR (i.e., 100%); Lysosomes at membrane of TH(+) neurons = 106 ± 5.0 mm^2^; Lysosomes in cytoplasm of TH(+) neurons = 111 ± 6.1 mm^2^; Total lysosomes in TH(+) neurons = 217 ± 7.4 mm^2^. Results are expressed as % of CTR. Each bar represents the mean ± SEM, Student t-test, *p < 0.05, (n = 4-6 / group).

### Proposed Mechanism of Action of AZP2006

Fig 8 illustrates a proposed mechanism of action for AZP2006, based on the experimental evidence presented in this study. (**1**) PGRN associates with PSAP in the lysosome; AZP2006, because of its strong lysosomal tropism, accumulates in lysosomes and binds to the PGRN/PSAP complex (as previously shown, 15). (**2**) This binding stabilizes the PGRN/PSAP complex, protecting it from proteolytic cleavage, therefore increasing their levels and maintaining their enzymatic activity, enhancing lysosomal function and helping to restore lysosomal homeostasis. (**3**) The AZP2006-PGRN/PSAP complex is transported to the plasma membrane via lysosomal exocytosis, facilitating the release of lysosomal contents into the extracellular space. (**4**) Due to AZP2006 preventing the processing/degradation of PGRN and PSAP, their levels would be increased extracellularly, thereby contributing to AZP2006’s neuroprotective effects. (**5**) A similar mechanism may also occur in microglial cells, increasing PGRN levels within these cells (**6**). Since PGRN is known to inhibit chronic inflammatory responses, AZP2006 may reduce cytokine release by activated microglial by increasing PGRN levels in microglial cells.

**Fig 8:**
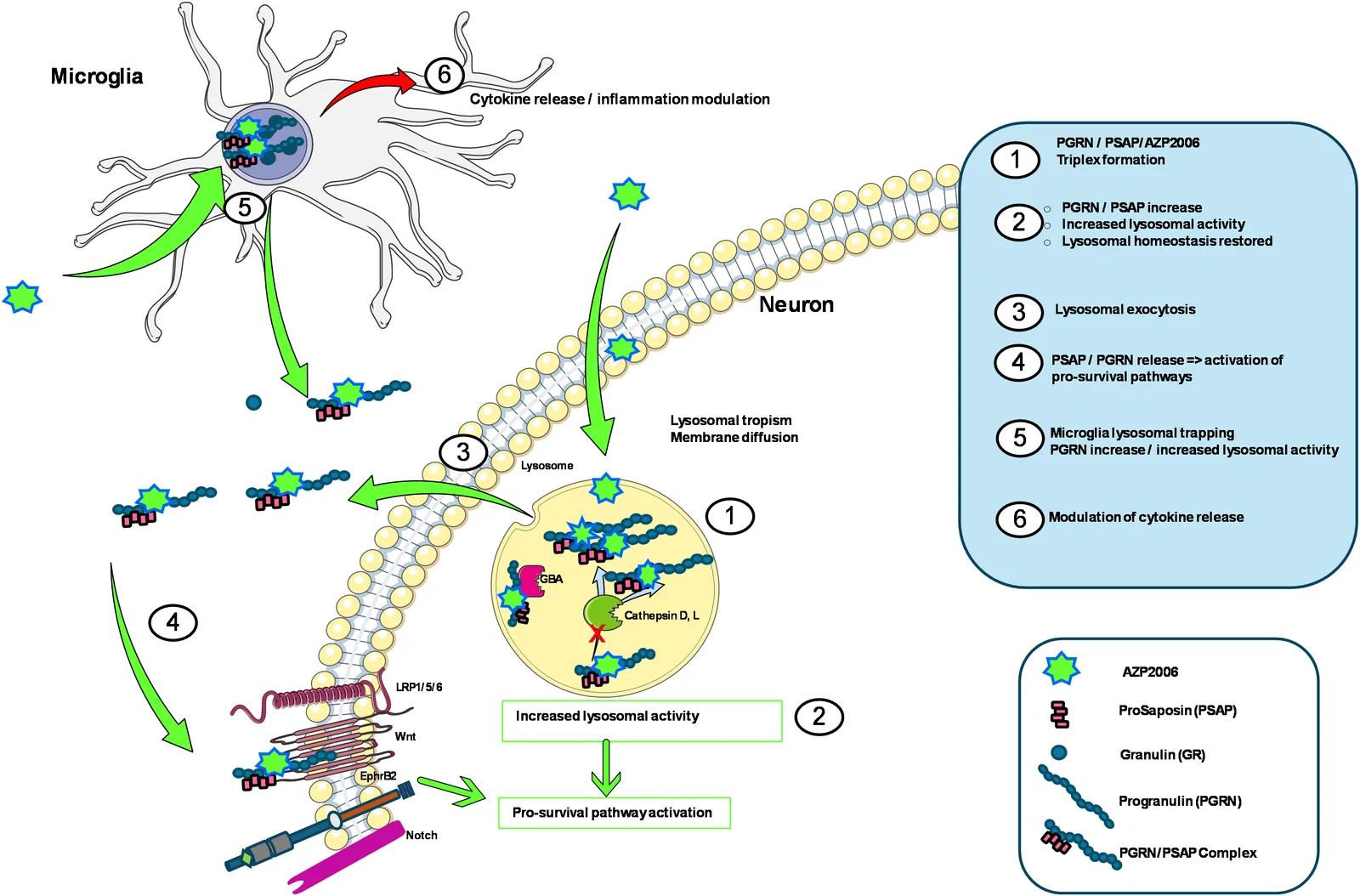
Schema of hypothesized AZP2006 mechanism of action.

## Discussion

In this study, AZP2006 (a lysosomotropic compound, see Callizot et al., 2021) protected dopaminergic neurons in both *in vitro* and *in vivo* models of PD based on mitotoxin and α-syn PFF-mediated toxicity. AZP2006 reduced α-syn accumulation and alleviated lysosomal stress, while restoring lysosomal function following injury. Furthermore, its mechanism of action in these models was found to involve PGRN, a key regulatory protein of lysosomal activity. These findings reveal that the neuroprotective effects of AZP2006 are mediated through its interaction with a lysosomal-associated target.

Lysosomal dysfunction has been implicated in PD pathology, making it a promising target for restoring protein clearance and neuronal health [31]. The homeostasis of the lysosome is tightly regulated and its disruption can drive the accumulation of aggregation-prone proteins, lipids and damaged organelles [4]. Given that impaired protein homeostasis is a central mechanism in PD, lysosomal dysfunction, including morphological and numerical abnormalities, has been frequently documented in both genetic and sporadic forms of PD [32]. Furthermore, lysosomal proteins such as lysosome-associated membrane protein 1 (LAMP1), cathepsin D (CTSD), and heat shock protein 73 (HSP73) are reduced in dopaminergic neurons of people with PD [32–34]. Identifying a therapeutic strategy to restore or improve lysosomal function may counteract synucleinopathies.

In previous work, AZP2006 has shown its ability to target lysosomal compartments and bind to the PGRN/PSAP complex [26]. Extensive research has shown that PGRN and PSAP play a crucial role in lysosomal homeostasis, notably by regulating enzymatic function [17,35,36].

Here, we showed that AZP2006 concentration-dependently abolished α-syn accumulation in the cytoplasm of primary dopaminergic neurons injured with an α-syn preparation containing PFF. This effect was linked to decreased lysosomal stress, reduced autolysosomes and autophagosomes, and an improved lysosomal function, indicating restored autophagic flux and enhanced protein clearance. Building on previous findings that AZP2006 induces its neuroprotective effects through increasing the availability of PGRN in pre-clinical models of Alzheimer’s disease [26], we have identified a similar mechanism of action in models of PD. In the absence of PGRN – either by using an antibody targeting PGRN in primary neuronal culture or through its genetic knockout in SH-SY5Y cells – the neuroprotective effects of AZP2006 are totally abolished. From these results, we can postulate that AZP2006 targets the endosome-lysosome pathway (likely due to its high lipid permeability) through binding to the PGRN/PSAP complex [26]. In doing so, it acts as a molecular chaperone, inhibiting their cleavage into granulin and saposin (see proposed mechanism of action, Fig 8). By maintaining the PGRN/PSAP complex, AZP2006 supports enzymatic functioning of the lysosome following α-syn toxicity.

Our *in vitro* findings were well translated into an *in vivo* model of PD whereby aged mice were injured with intra-nigral injections of α-syn PFF [28] which resulted in a significant degeneration of dopaminergic neurons of the SNpc. This degeneration was associated with accumulation of α-syn within dopaminergic neurons and microglial activation (S5 Fig, 28). Daily AZP2006 treatment (from the day of lesion, for 5 weeks) significantly preserved dopaminergic neurons and completely prevented pathological α-syn accumulation in their cytoplasm. Under physiological conditions, α-syn turnover is regulated by intracellular degradation systems, such as the ubiquitin-proteasomal system and the autophagy-lysosomal pathway. However, pathological forms of α-syn are not adequately degraded by these mechanisms, leading to their accumulation. These present findings indicate that AZP2006 treatment improves the functioning of neuronal degradation systems.

An additional contributing factor to PD pathology is neuroinflammation, driven by microglia. In our *in vitro* and *in vivo* models of PD, AZP2006 reduced microglial activation, induced by α-syn PFF toxicity. The neuroprotective effects of AZP2006 was certainly led by a direct effect on microglia. PGRN is secreted by microglia and PGRN deficiency in microglia activates NF-κB signaling and promotes the release of pro-inflammatory cytokines [37]. PGRN reduction in microglia alone is sufficient to recapitulate inflammation and lysosomal dysfunction [38]. Interestingly, we have shown that AZP2006 increases microglial PGRN in an *in vitro* model of Alzheimer’s disease, where a culture of human microglia cells were injured with Aβ_1–42_ (S6 Fig). These results, together with current and previous data, suggest that AZP2006 can restore and increase PGRN levels causing a direct effect on microglial activation.

Further research is required to better understand the precise mechanism of action of AZP2006. While its relationship to the lysosome is well-established, and its close relationship with PGRN and PSAP is clear, AZP2006’s direct impact on lysosomal functionality and homeostasis remains to be further investigated. Lysosomal quality control mechanisms are generally activated in response to any event characterized by membrane damage, lysosomal swelling, and the release of lysosomal luminal content into the cytosol, with possible uncontrolled breakdown of biomolecules. A variety of exogenous and endogenous factors can induce lysosomal membrane permeabilization, such as: lysosomotropic agents, compounds entrapped in the lysosomes after protonation, ROS, the apoptotic regulator Bcl-2-like protein 4, and infectious pathogens [39]. Lysosomal membrane permeabilization may also occur in response to neurotoxic events in neurodegenerative disease. Lysosomal quality control consists of multiple pathways dedicated to lysosomal repair, clearance (lysophagy), exocytosis, and biogenesis (lysogenesis). We showed that in basal conditions, AZP2006 (in parallel to its effects on PGRN/PSAP), can increase the proportion of lysosomes at the plasma membrane. We hypothesize that AZP2006 increases basal lysosomal efficiency by inducing a lysosomal flush that facilitates the release of PGRN and PSAP outside of the cells. Once released, these factors may engage their respective targets (i.e., Wnt, EphrB…see Fig 8), activating survival signaling pathways that could provide an additional explanation to AZP2006’s neuroprotective effects.

It should be noted that targeting a stressed lysosome does not have the same effect as targeting a lysosome under normal conditions. In a diseased environment, a large accumulation of lysosomes, filled with cellular waste (e.g., aggregated α-syn) is found, as the system attempts to achieve homeostasis. As we have described, AZP2006 improved lysosomal function through its interaction with PGRN. However, its activity on a functioning lysosome, in basal conditions, could result in lysosomal stress. As previously mentioned, lysosomal homeostasis is tightly regulated and if disrupted, such as through overactivation of a lysosomal pathway, may lead to negative cellular effects. We hypothesize that in basal conditions, AZP2006 would induce an increase in empty lysosomal vesicles **—** future studies should identify the lysosomal load following AZP2006 application, in basal versus disease conditions. Based on lysosomal biology, applying lysosomotropic agents in the presence of lysosomal stress and dysfunction will likely result in improved outcomes, compared to in a healthy, functioning environment.

This study provides the first demonstration of the neuroprotective effects of AZP2006 across multiple preclinical models of Parkinson's disease. We paid particular attention to the effects of AZP2006 on the lysosome, as its tropism for this organelle seems to be a key component of its neuroprotective mechanism. Given the well-established role of lysosomal homeostasis in neurodegeneration, targeting lysosomal quality control seems to be an attractive and effective strategy to address proteinopathies and neurodegenerative diseases. AZP2006 has already completed a successful Phase 2a trial in PSP patients [27,40] and a larger multicentric basket Phase 2b trial is planned in the US for the same indication. The evidence from this study, and the resulting insights into its mechanism of action, open new perspectives for the development of AZP2006 as a disease-modifying therapy for PD.

## Supporting information

S1 Fig Segmentation analysis for immunocytochemistry images from *in vitro* models of PD. Cell segmentation to separate the nuclear and cytoplasmic compartments which were then used to assess the area of markers, e.g., within the cytoplasmic compartment (A). Neurite segmentation in TH(+) neurons (red), observed in green (B). PSD95(+) puncta (green) in TH(+) neurites (red) (C).
(TIF)

S2 Fig Overview of the methods applied to investigate the AZP2006 in Parkinson’s Disease models. (1) Primary rat mesencephalic cultures (+/- microglia) were used to investigate the neuroprotective effects of AZP2006 following injury with dopaminergic mitotoxins (6OHDA (48h) MPP^+^ (48h), rotenone (24h) and α-syn PFF toxicity (48 or 96h). (2) SH-SY5Y cells + /- PGRN knock-out were applied to understand the mechanism of action of AZP2006 and its effects on lysosomal function following MPP^+^ (48h) or α-syn PFF toxicity (72 h). (3) Aged mice were administered with α-syn PFF into the substantia nigra pars compacta (SNpc) and the neuroprotective effects of daily administration (p.o.) of AZP2006 (for 5 weeks from the day of lesion) were assessed.
(TIF)

S3 Fig Effects of AZP2006 on TH(+) primary mesencephalic neurons injured with 6OHDA (20 μM) for 48h in the presence of an antibody unrelated to PGRN (anti-CD206 antibody). ICC analysis of cultures show the total number of TH(+) neurons (A), the length of their neurite network (B). Raw values for control (CTR) (i.e., 100%); TH(+) neurons = 35 ± 1; neurite network = 2137 ± 96 µm. Each bar represents the mean ± SEM, one-way ANOVA followed by Fisher’s test. ^####^p < 0.0001 from CTR and **p < 0.01 and ****p < 0.0001 from 6OHDA (n = 5–6 / group).
(TIF)

S4 Fig Effects of α-syn preparation infusion in the SNpc of aged mice for up to 5 weeks. IHC analysis shows the total number of TH(+) neurons (A), α-syn accumulation in TH(+) neurons (B) and the number of Iba1(+) microglia in SNpc (C) expressed as % of sham. Raw values for sham (i.e., 100%); TH(+) neurons = 70 ± 2.0; α-syn area/TH(+) neuron = 1229 ± 47 µm^2^; Iba1(+) cells = 249 ± 7.1. Each bar represents the mean ± SEM, one-way ANOVA followed by Fisher’s test. ^#^p < 0.05, ^##^p < 0.01, ^###^p < 0.001 or ^####^p < 0.0001 from sham (n = 7 mice / group).
(TIF)

S5 Fig Effect of neuronal survival following MPP^+^ (500 nM or 1 mM, 48h) to wild-type (WT) or PGRN knock-out (KO) SH-SY5Y cell cultures. ICC analysis of cultures show the total number of TH(+) neurons, expressed as % of control (CTR). Raw values for CTR (i.e., 100%); cells/field WT SH-SY5Y 142 ± 44. Each bar represents the mean ± SEM, two-way ANOVA followed Fisher’s LSD test. ***p < 0.001 and ****p < 0.0001 from CTR (respectively) (n = 4–6 / group).
(TIF)

S6 Fig Effect of AZP2006 on PGRN levels in the supernatant of a culture of human microglial clone 3 cell line (HMC3) injured with Aβ_1–42_ (5 μM,24h). The level of PGRN in the culture medium was determined by an enzyme-linked immunosorbent assay (ELISA). Data are expressed as % of control (CTR). Raw values for CTR (i.e., 100%); PGRN level 1 ± 1.99 ng/mL. Each bar represents the mean ± SEM, one-way ANOVA followed by Fisher’s test. ^*^p < 0.05 from control (n = 4–6 / group).
(TIF)

S1 File Raw data AZP2006 in Parkinsons disease models. (XLSX)
