# Supplementary material for: AZP2006 in Parkinson’s disease models: A promising new neuroprotective treatment involving lysosomal homeostasis and the Progranulin/Prosaposin complex
Source: PLoS One. 2026 Sep 30;21(9):e0359269. doi: 10.1371/journal.pone.0359269 (PMC13626330; doi:10.1371/journal.pone.0359269)
Supplement: Supplementary file 1 [file pone.0359269.s009.docx]

Dear Editor,

Thank you for reviewing our manuscript. We would like to thank the reviewers for their hard work and their constructive feedback on our manuscript. Thanks to the extension time, we have been able to conduct a significant number of additional analyses and experiments that have greatly improved the quality of the manuscript.

Please find the manuscript with the added results and the requested clarifications and explanations. You will also note that we have added two co-authors to the list of authors who contributed significantly to improving the manuscript and who participated in the additional analyses.

Below are the responses to each comment from the editor and reviewers.

**Editor comments**

1) Please clarify where data are housed - please note: Stating ‘data available on request from the author’ is not sufficient. If your data are only available upon request, select ‘No’ for the first question and explain your exceptional situation in the text box.

An Excel file containing all the raw data from each experiment is now included with the document to make it freely available.

2) Please clarify the timeline of lysosome exocytosis (96 hours treatment began when?). "For the study of lysosome exocytosis, cells were treated with 100 nM AZP2006 for 96 hours, with a second administration on day 8."
How long were cells treated for, in total?

This is now clearly written in the results section for all experiments. For example, in the sentence mentioned above; AZP2006 was added on day 6 and on day 8 of culture, on day 10, the culture was stopped.

3) Please provide RRIDs for all cell lines and antibodies, if possible.

This has been added in Table 1 and Table 2 for antibodies and cell lines added within the text (methods section), where possible.

4) Please clarify the number of biologic replicates as well as technical replicates for all in vitro work.

Each figure reports the technical replicates for the vitro work. A line has been added to the methods section stating ‘Each experiment consists of one culture (3 immunocytochemistry (ICC) markers + Hoechst stain per culture) and numbers are reported as biological replicates (n = 4-6 / group).’

5) Please clarify the power associated with the small in vivo group sizes, for the endpoints used.

This is now added under the section ‘Intranigral injection of a-syn preparation in mice and treatments’ stating the following:

‘Based on previous work in this model (28), power analysis (effect size = 2; alpha = 0.05; power = 0.9) indicated that for histological analysis, n = 7 mice / group are necessary to detect a statistical difference between two groups.’

6) Please clarify the pixel resolution for lysosome confocal imaging.

A new set of images is now provided with a better resolution. Additionally, this detail is added under the ‘Immunostaining and automated image analysis’, stating ‘pixel resolution = 71 nm x 71 nm per pixel’.

7) For all ANOVAs, please provide ANOVA data, e.g., numerator + denominator with F value. Please justify the use of Fisher’s LSD post-hoc test, which does not correct for multiple comparisons.

F values have been added to the excel spreadsheet containing all the raw values. Fisher’s LSD post-hoc test has been used because all concentrations of AZP2006 tested are independent of each other and we are not interested in the interactions between concentrations. Therefore, Fisher’s LSD test was used to understand the effect of AZP2006 (single concentration), compared to control and injured condition.

5. Review Comments to the Author

Please use the space provided to explain your answers to the questions above. You may also include additional comments for the author, including concerns about dual publication, research ethics, or publication ethics. (Please upload your review as an attachment if it exceeds 20,000 characters)

Reviewer #1: This manuscript presents a comprehensive preclinical study investigating the neuroprotective potential of AZP2006 (Ezeprogind) in several complementary Parkinson’s disease (PD)-like models, including primary neurons, SH-SY5Y cells, PGRN knockout systems, and an in vivo α-syn PFF model in aged mice. The work is timely and relevant, as it addresses the crucial link between lysosomal dysfunction, α-synuclein aggregation, and neurodegeneration, while also highlighting the role of the PGRN/PSAP complex in mediating lysosomal homeostasis. The breadth of models used and the mechanistic insights provided represent a significant strength of the study.
However, there are several points that require clarification and additional discussion. Some of these comments reflect missing experimental evidence that would strengthen the proposed mechanism of action, while others relate to presentation and clarity.

1. The authors propose that AZP2006 exerts its neuroprotective effects through binding and stabilization of the PGRN/PSAP complex. However, the study does not directly assess PGRN or PSAP protein levels or their stabilization in the presented models.

The reviewer is correct; we demonstrated the link between AZP2006 and the PGRN/PSAP complex in our previous publication (Callizot et al. 2021) and the increase of PGRN and PSAP have been demonstrated in human (Corvol et al., 2025). In this work, we demonstrated the importance of PGRN through our experiments using KO cells and progranulin-neutralizing antibodies. Additionally, in the supplementary information (S6 Fig.) we show that AZP2006 increases the release of PGRN in injured cells.

2. The study evaluates LAMP2 as a lysosomal marker, which is informative, but insufficient to capture the complexity of lysosomal function. Key downstream enzymes such as cathepsins (Cathepsin D) are not analyzed, despite their relevance to α-syn degradation.

Correct, additional experiments have been performed and included into the manuscript (Fig. 2a, H) showing the effect of AZP2006 on lysosomal function, using PhenoVue 577 lysosomal stain. AZP2006 was able to restore the acidic pH in SH-SY5Y cells injured with α-syn.

3. The introduction provides a broad overview of lysosomal dysfunction in PD but becomes somewhat overloaded and less focused. A more concise introduction centered on PGRN/PSAP biology, its relevance to PD, and how AZP2006 builds upon this would improve readability.

The introduction has been rewritten for more clarity following the reviewer’s advice and bibliography updated accordingly.

4. The study makes use of numerous models (mesencephalic neurons, SH-SY5Y cells, PGRN KO, α-syn PFF in mice, etc.), which can be difficult for the reader to follow. A schematic summarizing the experimental design across models would greatly aid comprehension.

A comprehensive schematic has been included in the supplementary information (S2 Fig.) for clarity.

5. The reduction of microglial activation is an important observation. However, it remains unclear whether this is a direct effect of AZP2006 on microglia or secondary to reduced α-syn toxicity in neurons.

In a related in vitro model of neurotoxicity, we demonstrate the direct effect of AZP2006 on microglial cells (human microglial cell line (HMC3)). Previously published data (Callizot et al. 2021) showed the effect of AZP2006 on the reduction of microglial activation. Additionally, we find that, in this model, AZP2006 is associated with an increased PGRN production. These important data have been added in supplementary figure 6 (S6 Fig.). Further information on the effect of AZP2006 on microglia has been included in the discussion section.

6. In statistical analysis more details could be provided: clarification on whether normality was tested, and multiple comparison corrections.

This information has been included in the methods section under statistical analysis. ‘Data are expressed as mean ± SEM (standard error of the mean). Shapiro-Wilks normality test was run for each data set. As the data was normally distributed, statistical analysis was performed by a one-way ANOVA or two-way ANOVA, followed by a Fisher’s LSD test, or Student *t*-test, as appropriate, using GraphPad Prism (version 10).’

7. While the discussion emphasizes lysosomal restoration as a key mechanism of AZP2006, the study does not provide direct functional evidence for this claim. Measures such as cathepsin activity, lysosomal pH, or other functional lysosomal markers are not reported. Therefore, the conclusion regarding lysosomal recovery is largely inferential and would benefit from additional functional validation.

Additional experiments have been performed and included into the manuscript (Fig. 2a, H) showing the effect of AZP2006 on lysosomal function, using PhenoVue 577 lysosomal stain. Increased staining is indicative of increased function as it represents increased pH. AZP2006 was able to restore the acidic pH in SH-SY5Y cells injured with α-syn. We agree that this could further confirmed by analysis of cathepsin activity in future work.

Reviewer #2: In the manuscript “AZP2006 in Parkinson’s Disease Models: A Promising New Neuroprotective Treatment Involving Lysosomal Homeostasis and the Progranulin/Prosaposin Complex” Callizot and colleagues present data on the neuroprotective action of the small compound AZP2006 in different Parkinson’s disease models. Employing primary neuronal cultures from mice and rats and a newly generated PRGN-KO SY5Y cell line, the compound was tested in different stress models related to Parkinson’s disease, including 6-OHDA and rotenone and the exposure with a-synuclein preparations. In addition, oral application of the compound reduced the toxic effect of stereotactic a-synuclein injections in the substantia nigra pars compacta. On the mechanistic side, the incubation of PRGN antibodies and the analysis of SY5Y PRGN-KO cells imply a central role of PGN in the protective action of AZP2006. These data as well as previous work of the group in other neurodegenerative disorders foster the view that the AZP2006-PRGN interaction supports lysosomal system function which seems to be beneficial under different stress conditions.
Despite there is limited new information related to the mode of action of AZP2006, it is an interesting dataset for the community as the compound was tested in a new disease condition. Nonetheless, several aspects need to be addresses.

Major points:

1) As described in the Method section, almost all data were achieved by (semi)automated image acquisition and processing. Please provide sample images for the conditions, best as a supplementary figure, particularly, but not exclusively, for the analysis of the synapses and a-syn aggregation. For the latter, are there punctae, as the wording “a-syn aggregation” imposes, or has been the “area of cytoplasmic a-syn” been analyzed as described in the legend. Please rephrase!

The segmentation processes used for these readouts have been included in supplementary figure 1 (S1 Fig.). Additionally, the wording has been altered and is consistent throughout the manuscript (including figure titles) as ‘α-syn accumulation’ or ‘area of cytoplasmic α-syn’.

2) There seems to be a problem when uploading the files. When clicking the supplemental material on the last page of the manuscript PDF provided for review, only one figure is visible. Unfortunately, the characterization of the a-syn preparations is missing.

All figures have been provided and uploaded. The characterization of the α-syn preparation has been thoroughly described in Henriques *et al* 2022 (reference 28).

3) The authors analyzed neuron number as well as the neurite network. Has the length of the neurite network been normalized for the neuron numbers?

As the analysis is automatic, the whole well is analysed and 20 or 9 images (fields of view) were analysed per well, with 6 wells per condition. The segmentation is based on detection of TH(+) neurons and its associated neurites. The length of the neurite network is the averaged (mean) total neurite length per field of view, with only those emanating from a confirmed TH (+) neuron included.

4) Fig 1a E-H: PRGN antibodies were applied to the medium. Please detail when the antibody was applied: at the same time with the compounds or as a pre-treatment. Furthermore, seemingly the antibodies have neither an effect on all measures documented when applied alone nor do they result in a more severe phenotype. Please explain why an inactivation of extracellular (and potentially intracellular) PRGN has no effect. In addition, the authors do not provide an appropriate control with an unrelated antibody.

New experiments have been conducted with an antibody (anti-CD206) that is unrelated to any cellular or subcellular targets within the culture or the proposed mechanism of AZP2006, as a control. These results can be found in supplementary figure 3 and show that the effect of AZP2006 was unaffected in the presence of an antibody unrelated to PGRN.

5) Fig 1b and Fig 2a: The authors analyzed the ratio of LAMP2 and TH. Is this analysis solely based on areas and/or intensities? Furthermore, the authors report an accumulation of autophagosomes based on the area of LC3B with TH cells. LC3B area is not to the sufficient measure to determine autophagosomes or autolysosomes as it is soluble within the cell when unlipidated. Please provide data on LC3B-positive punctae with or without LAMP2 to determine autophagosomes and autolysosomes, respectively. Having said this, several publications report a direct impact of PRGN on autophagic flux. It would certainly strengthen the manuscript and add mechanistic insight on the action of AZP2006, when the authors provide evidence showing the impact of the compound on autophagic flux.

LAMP2 evaluation was measured as vesicle area. Additionally, LC3b vesicle area was performed (called vesicle area, this is now better explained in the method section and segmentation found in S1 Fig). LC3b vesicles were identified as individual vesicles with a determined max/min size, which takes into consideration its proximity to other identified positive vesicles, whereby those in too close proximity are excluded, therefore it’s the number of LC3b(+) vesicles within the zone of analysis.

6) Fig 3: The effect of the oral application of AZP2006 starting at the time of stereotactic a-syn injection is intriguing. The group published previously that mice treated in a similar way, showed motor deficits. Does AZP2006 restore motor function?

The initial aim of this study was to investigate the putative effect of AZP2006 on histological readouts. Following power analysis, only 7 animals per groups have been included. For behavioral readouts, n = 14 are required to see an effect between groups. In previous publications, appropriately powered groups were used to assess the effect of AZP2006 on motor function and co-ordination. Behaviour data were recorded in these mice but due to the inappropriate power of the study for behavioural assessment, no data were included. Below you will find data on the effect of AZP2006 on motor function and co-ordination in the ladder test, which assess the number of errors whilst crossing a ladder with uneven rungs. We know in this model at this time point there is little to no behaviour phenotype with α-syn only, the model requires more time to show a behavioural effect. We have shown this in our model with α-syn + CBE, which increases disease progression and a motor deficit is observed (Henriques et al., 2022). Despite this, there is a modest trend to a reduced number of errors in the presence of AZP2006 in this model. Future studies should assess the effect on AZP2006 on motor function within in vivo models of Parkinson’s disease.

**Figure 1. The effect of AZP2006 on motor co-ordination in aged mice injured with α-syn preparation in the SNpc and treated with AZP2006 for 5 weeks.** The ladder test was used to assess motor function and co-ordination. The mice cross a ladder with un-even rungs and the number of errors (slips) are automatically detected. A mean of 3 runs are recorded (n = 7 mice / group). Data are presented as mean ± SEM. Data were tested for normality and were not found to be normally distributed. A Kruskal-Wallis test followed by Fisher’s LSD test was used to compare to the α-syn + vehicle group. No significance was found between groups (p<0.05).

Nonetheless, it seems that the selection of images (B) does not reflect the quantification (C-E). As an example, the number of microglia is massive without treatment, but there are only about 30% more microglia in numbers compared to the other conditions. Please provide more representative images. Furthermore, it looks like that the center panel in B shows the injection site. Is this true also for the other panels? Please provide lower power images to visualize the area analyzed. One more note, please change the wording “a-syn aggregation” as seemingly the area relative to TH neurons was determined, but not aggregates (see above).

New representative images have been added to Figure 3B to best represent the data. The images are representative of the SNpc region. Additionally, the wording has been changed to ‘α-syn accumulation within TH neurons’, to better reflect the analysis.

7) Fig 4: These data clearly show that the activity of AZP2006 is directly linked to the presence of PRGN within cells. Please explain in the text, why SY5Y wildtype cells were treated with different MPP concentrations as the PRGN KO cells. Please provide information, if the PRGN KO cells differentiate the same way as the wildtype SY5Y cells.

Additional information has been described within the text and data added to supplementary figure 6 which shows the concentration-response curve of WT and PGRN KO cells. The concentration discrepancy is based on the different sensitivity observed between KO and WT. The goal of this experiment was to achieve a similar level of injury between the two cell types. This is now explained in the text. ‘Concentrations of MPP+ were chosen to achieve the same level of neuronal survival between WT and PGRN KO cell cultures after intoxication (S5 Fig.).’

8) Fig 5: The data provided here are weak and do not show convincingly an alteration of the subcellular distribution of lysosome upon AZP2006 treatment. (i) First of all, having seen the initial data showing the reduction of stress-induced LAMP2-positive area upon AZP2006 treatment it is counterintuitive that AZP2006 alone induces an increase in LAMP2-positive punctae, although non-significant. Please discuss why this might be the case.

(ii) To determine a membrane-associated localization, cells need to be co-stained with a bonafide membrane protein. (iii) It seems that the total number and the membrane and cytosolic lysosome numbers rise about the same level. Nonetheless, only the numbers in C are significant. This is seemingly based on a lower SEM under this condition. Therefore, the conclusions drawn out of this dataset and discussed in the Discussion section are not fully supported.

This is an important point and this is now discussed in the discussion section of the main text. Acting on a stressed lysosome does not have the same effect as acting on an un-stressed lysosome. AZP2006 improves lysosomal function through its action on PGRN. However, its action on a fully functional lysosome can only result in stress in the long term, as it will disrupt the normal homeostasis

Minor points: Overall, there are several inconsistencies and inaccuracies within the manuscript. A couple of them are listed below, but please revise carefully!

Numerous sections have been re-written for more clarity. Additional information, new analysis and experiments have been included. We hope that will improve the quality of the manuscript.

9) Fig 1a, legend: A-D is without the PGRN antibody and E-H is with the antibody

This has been updated.

10) There is a mixup of legends for Fig 2a and Fig 2b

This has be corrected.

11) Fig 3A: It is not shown in Fig 3A that “Daily administration of AZP2006 for 5 weeks post-lesion” (p 14 2. paragraph). Please add the line.

Now included in the figure legend of Figure 3A.

6. PLOS authors have the option to publish the peer review history of their article ([what does this mean?](https://fra01.safelinks.protection.outlook.com/?url=https%3A%2F%2Ftrack.editorialmanager.com%2FCL0%2Fhttps%3A%252F%252Fjournals.plos.org%252Fplosone%252Fs%252Feditorial-and-peer-review-process%2523loc-peer-review-history%2F1%2F010f019a603a7296-51177ebd-1ad5-4ad0-933e-26c36195453e-000000%2F43GDO1XPb5OUc194jdFwbTOyrPvM2kg07xqx6w6GYa0%3D236&data=05%7C02%7Cgrace.flower%40neuro-sys.com%7C3f7cf38f2f5e484dabf408de255c999f%7C93e9c267c9544697af7ab58941c75b6f%7C0%7C0%7C638989276802916123%7CUnknown%7CTWFpbGZsb3d8eyJFbXB0eU1hcGkiOnRydWUsIlYiOiIwLjAuMDAwMCIsIlAiOiJXaW4zMiIsIkFOIjoiTWFpbCIsIldUIjoyfQ%3D%3D%7C0%7C%7C%7C&sdata=xPZ6F3uy0vJ31FZM9Kur%2FCQpPl3pFjP1k%2B7Y8K1A1yE%3D&reserved=0)). If published, this will include your full peer review and any attached files.


If you choose “no”, your identity will remain anonymous but your review may still be made public.


Do you want your identity to be public for this peer review? For information about this choice, including consent withdrawal, please see our [Privacy Policy](https://fra01.safelinks.protection.outlook.com/?url=https%3A%2F%2Ftrack.editorialmanager.com%2FCL0%2Fhttps%3A%252F%252Fwww.plos.org%252Fprivacy-policy%2F1%2F010f019a603a7296-51177ebd-1ad5-4ad0-933e-26c36195453e-000000%2FTcZ41X0MpvHbGEcBYwoonruIIMCvO0G9Cn4B4wUHOP0%3D236&data=05%7C02%7Cgrace.flower%40neuro-sys.com%7C3f7cf38f2f5e484dabf408de255c999f%7C93e9c267c9544697af7ab58941c75b6f%7C0%7C0%7C638989276802931796%7CUnknown%7CTWFpbGZsb3d8eyJFbXB0eU1hcGkiOnRydWUsIlYiOiIwLjAuMDAwMCIsIlAiOiJXaW4zMiIsIkFOIjoiTWFpbCIsIldUIjoyfQ%3D%3D%7C0%7C%7C%7C&sdata=06h0NO05N%2BfvoqeFc%2FUAJV5IWioDsDDr7uRMGBDrg74%3D&reserved=0).

Reviewer #1: No

Reviewer #2: No

Thank you very much for taking the time to review our paper,

Yours sincerely,

Georgia Culley
