# Supplementary material for: AZP2006 in Parkinson’s disease models: A promising new neuroprotective treatment involving lysosomal homeostasis and the Progranulin/Prosaposin complex
Source: PLoS One. 2026 Sep 30;21(9):e0359269. doi: 10.1371/journal.pone.0359269 (PMC13626330; doi:10.1371/journal.pone.0359269)
Supplement: Supplementary file 2 [file pone.0359269.s010.docx]

Dear Editor,

Thank you for the opportunity to revise and resubmit our manuscript. We are grateful to you and the reviewers for the careful evaluation of our work and for the constructive and insightful comments.

In response to the additional points raised by Reviewer 2, we have modified the text of the manuscript and improved its clarity. Please find enclosed the revised manuscript, which includes the requested clarifications and corrections. Below, we provide a detailed, point-by-point response to Reviewer 2's comments.

Additional points raised after resubmission (19-05-2026)

6. Review Comments to the Author

Reviewer #2: The revised version of the manuscript entitled “AZP2006 in Parkinson’s Disease Models: A Promising New Neuroprotective Treatment Involving Lysosomal Homeostasis and the Progranulin/Prosaposin Complex” by Callizot and colleagues had been substantially improved. A few comments are listed below:

1) Introduction: Page 2, end 2nd paragraph: please set “lysosomal-autophagic system” for “lysosomal-autophagy” system

Thank you, this has now been modified.

2) Introduction: Page 2, first sentence of 3rd paragraph: please check grammar

This has been modified to; “Lysosomal function depends on tightly regulated homeostasis mediated by lysosomal ion channels, which maintain the acidic pH necessary for lysosomal enzyme activity (4,5).”

3) Introduction: Page 4, first sentence: did you mean: ..therapy development…”

This has been modified to “AZP2006 is being developed as a therapy for tauopathies, including Progressive Supranuclear Palsy (PSP), where a Phase 2a trial (NCT04008355) demonstrated favorable clinical outcomes (27).”

4) Figure legends with cellular analysis: what does n = 4-6 / group mean? I guess, 4-6 independent experiments were included. How many cells were included per independent experiment?

We thank the reviewer for requesting clarification. Within one culture plate, 4–6 wells (biological replicates) were included per condition. Multiple fields of view were acquired from each well and analyzed automatically using the image-analysis pipeline. Data from all fields of view within a well were summed to generate a single value, such that each well represented one biological replicate. Depending on the assay, approximately 12, 600 total cells (or 19, 800 when microglial proliferation was enabled), including glial cells and non-TH(+) were analyzed per well, therefore 50, 000 – 75, 000 per condition (or 80, 000 – 120, 000 with microglial proliferation). Of these cells, a relatively small proportion were dopaminergic neurons. We have modified the figure legends to clarify the number of TH(+) cells per well as such; “expressed as % of control (CTR). Raw values for CTR (i.e. 100 %); sum TH(+) neurons per well, i.e. per biological replicate = 27 ± 1.0 (figure 1a).” Experiments performed on SH-SY5Y cells (figure 2a.H and figure 4) had a higher number of TH(+) cells and data was therefore provided as an average per image field rather than per well (this has been further clarified in the figure legends.

5) Page 16, last paragraph. Referencing of Fig 3 panels in the main text is not correct. Please re-check!

Thank you for pointing this out – the figure referencing has now been corrected.

Thank you very much for taking the time to review our publication,

Yours sincerely,

Georgia Culley
