# Supplementary material for: AZP2006 in Parkinson’s disease models: A promising new neuroprotective treatment involving lysosomal homeostasis and the Progranulin/Prosaposin complex
Source: PLoS One. 2026 Sep 30;21(9):e0359269. doi: 10.1371/journal.pone.0359269 (PMC13626330; doi:10.1371/journal.pone.0359269)
Supplement: Supplementary file 3 [file pone.0359269.s011.docx]

Dear Editor,

Thank you for the opportunity to revise and resubmit our manuscript. We are grateful to you for the careful evaluation of our work and for the constructive and insightful comments.

In response to the additional points raised by Reviewer 2 regarding the references in the main text for Fig 3, we have carefully reviewed the manuscript. The figure-panel references identified during the previous review round had already been corrected in our prior resubmission. However, to ensure that the reviewer's concern has been fully addressed, we have re-examined all references to Figure 3 throughout the manuscript and made an additional clarification by explicitly citing Figure 3B (representative images) within the text.

We have therefore resubmitted:

- A clean revised manuscript;
- A tracked-changes version highlighting the amendments made; and
- This response document.

Please find below our point-by-point response to the reviewer's comment.

**Reviewer #2**

**Comment:**

The revised manuscript by Callizot and co-workers has been significantly improved and all commwnts were adressed. Please correct the references for Fig 3 in the main text (e.g. p16). Fig 3 does not show panels F and G.

**Response:**

We thank the reviewer for this positive assessment of our manuscript.

We carefully reviewed all references to Figure 3 in the manuscript. The incorrect references to Figure 3 panels had already been corrected during the previous revision round. Nevertheless, to ensure complete clarity and address the reviewer's concern, we performed an additional review of all Figure 3 citations and added an explicit reference to Figure 3B (representative images) in the relevant section of the text.

We hope this clarification fully addresses the reviewer's comment.

We thank the Editor and Reviewer #2 for their time and consideration and look forward to the final evaluation of our manuscript.

**Yours sincerely,**

Georgia Culley
On behalf of all authors
