# Supplementary material for: AZP2006 in Parkinson’s disease models: A promising new neuroprotective treatment involving lysosomal homeostasis and the Progranulin/Prosaposin complex
Source: PLoS One. 2026 Sep 30;21(9):e0359269. doi: 10.1371/journal.pone.0359269 (PMC13626330; doi:10.1371/journal.pone.0359269)
Supplement: Peer review history

# Peer review: Decision Letter 0

**DOI:** 10.1371/journal.pone.0359269.r001

**Authors:** Miriam Hickey

7 Nov 2025

Dear Dr. Culley,

Thank you for submitting your manuscript to PLOS ONE. After careful consideration, we feel that it has merit but does not fully meet PLOS ONE’s publication criteria as it currently stands. Therefore, we invite you to submit a revised version of the manuscript that addresses the points raised during the review process.

Please submit your revised manuscript by Dec 22 2025 11:59PM. If you will need more time than this to complete your revisions, please reply to this message or contact the journal office at plosone@plos.org. When you're ready to submit your revision, log on to https://www.editorialmanager.com/pone/ and select the 'Submissions Needing Revision' folder to locate your manuscript file.

- A rebuttal letter that responds to each point raised by the academic editor and reviewer(s). You should upload this letter as a separate file labeled 'Response to Reviewers'.
- A marked-up copy of your manuscript that highlights changes made to the original version. You should upload this as a separate file labeled 'Revised Manuscript with Track Changes'.
- An unmarked version of your revised paper without tracked changes. You should upload this as a separate file labeled 'Manuscript'.

If you would like to make changes to your financial disclosure, please include your updated statement in your cover letter. Guidelines for resubmitting your figure files are available below the reviewer comments at the end of this letter.

If applicable, we recommend that you deposit your laboratory protocols in protocols.io to enhance the reproducibility of your results. Protocols.io assigns your protocol its own identifier (DOI) so that it can be cited independently in the future. For instructions see: https://journals.plos.org/plosone/s/submission-guidelines#loc-laboratory-protocols. Additionally, PLOS ONE offers an option for publishing peer-reviewed Lab Protocol articles, which describe protocols hosted on protocols.io. Read more information on sharing protocols at https://plos.org/protocols?utm_medium=editorial-email&utm_source=authorletters&utm_campaign=protocols.

We look forward to receiving your revised manuscript.

Kind regards,

Miriam A. Hickey, PhD

Academic Editor

PLOS ONE

**Journal Requirements:**

1. When submitting your revision, we need you to address these additional requirements.

Please ensure that your manuscript meets PLOS ONE's style requirements, including those for file naming. The PLOS ONE style templates can be found at

https://journals.plos.org/plosone/s/file?id=wjVg/PLOSOne_formatting_sample_main_body.pdf and

https://journals.plos.org/plosone/s/file?id=ba62/PLOSOne_formatting_sample_title_authors_affiliations.pdf

2. Thank you for stating the following financial disclosure:

Philippe Verwaerde, CEO of Alzprotect, was awarded a grant for the project entitled "Using AZP2006 (Ezeprogind) to Stabilize Progranulin – A Novel Therapeutic Strategy for GBA1-linked Parkinson’s Disease" by the  Michael J. Fox Foundation.

https://www.michaeljfox.org/grant/using-azp2006-ezeprogind-stabilize-progranulin-novel-therapeutic-strategy-gba1-linked

Please state what role the funders took in the study.  If the funders had no role, please state: "The funders had no role in study design, data collection and analysis, decision to publish, or preparation of the manuscript."

If this statement is not correct you must amend it as needed.

Please include this amended Role of Funder statement in your cover letter; we will change the online submission form on your behalf.

3. Thank you for stating the following in the Competing Interests section:

This work was funded by Alzprotect. Philippe Verwaerde is the CEO of Alprotect, the company that develops AZP2006.

We note that one or more of the authors are employed by a commercial company.

a. Please provide an amended Funding Statement declaring this commercial affiliation, as well as a statement regarding the Role of Funders in your study. If the funding organization did not play a role in the study design, data collection and analysis, decision to publish, or preparation of the manuscript and only provided financial support in the form of authors' salaries and/or research materials, please review your statements relating to the author contributions, and ensure you have specifically and accurately indicated the role(s) that these authors had in your study. You can update author roles in the Author Contributions section of the online submission form.

Please also include the following statement within your amended Funding Statement.

“The funder provided support in the form of salaries for authors, but did not have any additional role in the study design, data collection and analysis, decision to publish, or preparation of the manuscript. The specific roles of these authors are articulated in the ‘author contributions’ section.”

If your commercial affiliation did play a role in your study, please state and explain this role within your updated Funding Statement.

b. Please also provide an updated Competing Interests Statement declaring this commercial affiliation along with any other relevant declarations relating to employment, consultancy, patents, products in development, or marketed products, etc.

Within your Competing Interests Statement, please confirm that this commercial affiliation does not alter your adherence to all PLOS ONE policies on sharing data and materials by including the following statement: "This does not alter our adherence to  PLOS ONE policies on sharing data and materials.” (as detailed online in our guide for authors http://journals.plos.org/plosone/s/competing-interests) . If this adherence statement is not accurate and  there are restrictions on sharing of data and/or materials, please state these. Please note that we cannot proceed with consideration of your article until this information has been declared.

Please include both an updated Funding Statement and Competing Interests Statement in your cover letter. We will change the online submission form on your behalf.

4. In the online submission form, you indicated that “Data will be made available on request.”

All PLOS journals now require all data underlying the findings described in their manuscript to be freely available to other researchers, either a. In a public repository, b. Within the manuscript itself, or c. Uploaded as supplementary information.

This policy applies to all data except where public deposition would breach compliance with the protocol approved by your research ethics board. If your data cannot be made publicly available for ethical or legal reasons (e.g., public availability would compromise patient privacy), please explain your reasons on resubmission and your exemption request will be escalated for approval.

5. Please include your full ethics statement in the ‘Methods’ section of your manuscript file. In your statement, please include the full name of the IRB or ethics committee who approved or waived your study, as well as whether or not you obtained informed written or verbal consent. If consent was waived for your study, please include this information in your statement as well.

6. Please include captions for your Supporting Information files at the end of your manuscript, and update any in-text citations to match accordingly. Please see our Supporting Information guidelines for more information: http://journals.plos.org/plosone/s/supporting-information.

7. PLOS ONE now requires that authors provide the original uncropped and unadjusted images underlying all blot or gel results reported in a submission’s figures or Supporting Information files. This policy and the journal’s other requirements for blot/gel reporting and figure preparation are described in detail at https://journals.plos.org/plosone/s/figures#loc-blot-and-gel-reporting-requirements and https://journals.plos.org/plosone/s/figures#loc-preparing-figures-from-image-files. When you submit your revised manuscript, please ensure that your figures adhere fully to these guidelines and provide the original underlying images for all blot or gel data reported in your submission. See the following link for instructions on providing the original image data: https://journals.plos.org/plosone/s/figures#loc-original-images-for-blots-and-gels.

In your cover letter, please note whether your blot/gel image data are in Supporting Information or posted at a public data repository, provide the repository URL if relevant, and provide specific details as to which raw blot/gel images, if any, are not available. Email us at plosone@plos.org if you have any questions.

8. If the reviewer comments include a recommendation to cite specific previously published works, please review and evaluate these publications to determine whether they are relevant and should be cited. There is no requirement to cite these works unless the editor has indicated otherwise.

**Additional Editor Comments:**

Please address comments of Reviewer 1 and Reviewer 2 and also Editor comments, thank you.

**Editor comments**

1) Please clarify where data are housed - please note:

Stating ‘data available on request from the author’ is not sufficient. If your data are only available upon request, select ‘No’ for the first question and explain your exceptional situation in the text box.

2) Please clarify the timeline of lysosome excytosis (96 hours treatment began when?).

"For the study of lysosome exocytosis, cells were treated with 100 nM AZP2006 for 96 hours, with a second administration on day 8."

How long were cells treated for, in total?

3) Please provide RRIDs for all cell lines and antibodies, if possible.

4) Please clarify the number of biologic replicates as well as technical replicates for all in vitro work.

5) Please clarify the power associated with the small in vivo group sizes, for the endpoints used.

6) Please clarify the pixel resolution for lysosome confocal imaging.

7) For all ANOVAs, please provide ANOVA data, e.g., numerator + denominator with F value. Please justify the use of Fisher’s LSD post-hoc test, which does not correct for multiple comparisons.

Reviewers' comments:

**Comments to the Author**

1. Is the manuscript technically sound, and do the data support the conclusions?

Reviewer #1: Yes

Reviewer #2: Partly

**********

2. Has the statistical analysis been performed appropriately and rigorously?

Reviewer #1: Yes

Reviewer #2: Yes

**********

3. Have the authors made all data underlying the findings in their manuscript fully available?

The PLOS Data policy

Reviewer #1: Yes

Reviewer #2: No

**********

4. Is the manuscript presented in an intelligible fashion and written in standard English?

Reviewer #1: Yes

Reviewer #2: Yes

**********

**Reviewer #1:**  This manuscript presents a comprehensive preclinical study investigating the neuroprotective potential of AZP2006 (Ezeprogind) in several complementary Parkinson’s disease (PD)-like models, including primary neurons, SH-SY5Y cells, PGRN knockout systems, and an in vivo α-syn PFF model in aged mice. The work is timely and relevant, as it addresses the crucial link between lysosomal dysfunction, α-synuclein aggregation, and neurodegeneration, while also highlighting the role of the PGRN/PSAP complex in mediating lysosomal homeostasis. The breadth of models used and the mechanistic insights provided represent a significant strength of the study.

However, there are several points that require clarification and additional discussion. Some of these comments reflect missing experimental evidence that would strengthen the proposed mechanism of action, while others relate to presentation and clarity.

1. The authors propose that AZP2006 exerts its neuroprotective effects through binding and stabilization of the PGRN/PSAP complex. However, the study does not directly assess PGRN or PSAP protein levels or their stabilization in the presented models.

2. The study evaluates LAMP2 as a lysosomal marker, which is informative, but insufficient to capture the complexity of lysosomal function. Key downstream enzymes such as cathepsins (Cathepsin D) are not analyzed, despite their relevance to α-syn degradation.

3. The introduction provides a broad overview of lysosomal dysfunction in PD but becomes somewhat overloaded and less focused. A more concise introduction centered on PGRN/PSAP biology, its relevance to PD, and how AZP2006 builds upon this would improve readability.

4. The study makes use of numerous models (mesencephalic neurons, SH-SY5Y cells, PGRN KO, α-syn PFF in mice, etc.), which can be difficult for the reader to follow. A schematic summarizing the experimental design across models would greatly aid comprehension.

5. The reduction of microglial activation is an important observation. However, it remains unclear whether this is a direct effect of AZP2006 on microglia or secondary to reduced α-syn toxicity in neurons.

6. In statistical analysis more details could be provided: clarification on whether normality was tested, and multiple comparison corrections.

7. While the discussion emphasizes lysosomal restoration as a key mechanism of AZP2006, the study does not provide direct functional evidence for this claim. Measures such as cathepsin activity, lysosomal pH, or other functional lysosomal markers are not reported. Therefore, the conclusion regarding lysosomal recovery is largely inferential and would benefit from additional functional validation.

**Reviewer #2:**  In the manuscript “AZP2006 in Parkinson’s Disease Models: A Promising New Neuroprotective Treatment Involving Lysosomal Homeostasis and the Progranulin/Prosaposin Complex” Callizot and colleagues present data on the neuroprotective action of the small compound AZP2006 in different Parkinson’s disease models. Employing primary neuronal cultures from mice and rats and a newly generated PRGN-KO SY5Y cell line, the compound was tested in different stress models related to Parkinson’s disease, including 6-OHDA and rotenone and the exposure with a-synuclein preparations. In addition, oral application of the compound reduced the toxic effect of stereotactic a-synuclein injections in the substantia nigra pars compacta. On the mechanistic side, the incubation of PRGN antibodies and the analysis of SY5Y PRGN-KO cells imply a central role of PGN in the protective action of AZP2006. These data as well as previous work of the group in other neurodegenerative disorders foster the view that the AZP2006-PRGN interaction supports lysosomal system function which seems to be beneficial under different stress conditions.

Despite there is limited new information related to the mode of action of AZP2006, it is an interesting dataset for the community as the compound was tested in a new disease condition. Nonetheless, several aspects need to be addresses.

Major points

1) As described in the Method section, almost all data were achieved by (semi)automated image acquisition and processing. Please provide sample images for the conditions, best as a supplementary figure, particularly, but not exclusively, for the analysis of the synapses and a-syn aggregation. For the latter, are there punctae, as the wording “a-syn aggregation” imposes, or has been the “area of cytoplasmic a-syn” been analyzed as described in the legend. Please rephrase!

2) There seems to be a problem when uploading the files. When clicking the supplemental material on the last page of the manuscript PDF provided for review, only one figure is visible. Unfortunately, the characterization of the a-syn preparations is missing.

3) The authors analyzed neuron number as well as the neurite network. Has the length of the neurite network been normalized for the neuron numbers?

4) Fig 1a E-H: PRGN antibodies were applied to the medium. Please detail when the antibody was applied: at the same time with the compounds or as a pre-treatment. Furthermore, seemingly the antibodies have neither an effect on all measures documented when applied alone nor do they result in a more severe phenotype. Please explain why an inactivation of extracellular (and potentially intracellular) PRGN has no effect. In addition, the authors do not provide an appropriate control with an unrelated antibody.

5) Fig 1b and Fig 2a: The authors analyzed the ratio of LAMP2 and TH. Is this analysis solely based on areas and/or intensities? Furthermore, the authors report an accumulation of autophagosomes based on the area of LC3B with TH cells. LC3B area is not to the sufficient measure to determine autophagosomes or autolysosomes as it is soluble within the cell when unlipidated. Please provide data on LC3B-positive punctae with or without LAMP2 to determine autophagosomes and autolysosomes, respectively. Having said this, several publications report a direct impact of PRGN on autophagic flux. It would certainly strengthen the manuscript and add mechanistic insight on the action of AZP2006, when the authors provide evidence showing the impact of the compound on autophagic flux.

6) Fig 3: The effect of the oral application of AZP2006 starting at the time of stereotactic a-syn injection is intriguing. The group published previously that mice treated in a similar way, showed motor deficits. Does AZP2006 restore motor function? Nonetheless, it seems that the selection of images (B) does not reflect the quantification (C-E). As an example, the number of microglia is massive without treatment, but there are only about 30% more microglia in numbers compared to the other conditions. Please provide more representative images. Furthermore, it looks like that the center panel in B shows the injection site. Is this true also for the other panels? Please provide lower power images to visualize the area analyzed. One more note, please change the wording “a-syn aggregation” as seemingly the area relative to TH neurons was determined, but not aggregates (see above).

7) Fig 4: These data clearly show that the activity of AZP2006 is directly linked to the presence of PRGN within cells. Please explain in the text, why SY5Y wildtype cells were treated with different MPP concentrations as the PRGN KO cells. Please provide information, if the PRGN KO cells differentiate the same way as the wildtype SY5Y cells.

8) Fig 5: The data provided here are weak and do not show convincingly an alteration of the subcellular distribution of lysosome upon AZP2006 treatment. (i) First of all, having seen the initial data showing the reduction of stress-induced LAMP2-positive area upon AZP2006 treatment it is counterintuitive that AZP2006 alone induces an increase in LAMP2-positive punctae, although non-significant. Please discuss why this might be the case (ii) To determine a membrane-associated localization, cells need to be co-stained with a bonafide membrane protein. (iii) It seems that the total number and the membrane and cytosolic lysosome numbers rise about the same level. Nonetheless, only the numbers in C are significant. This is seemingly based on a lower SEM under this condition. Therefore, the conclusions drawn out of this dataset and discussed in the Discussion section are not fully supported.

Minor points:

Overall, there are several inconsistencies and inaccuracies within the manuscript. A couple of them are listed below, but please revise carefully!

9) Fig 1a, legend: A-D is without the PGRN antibody and E-H is with the antibody

10) There is a mixup of legends for Fig 2a and Fig 2b

11) Fig 3A: It is not shown in Fig 3A that “Daily administration of AZP2006 for 5 weeks post-lesion” (p 14 2. paragraph). Please add the line.

**********

what does this mean?). If published, this will include your full peer review and any attached files.

If you choose “no”, your identity will remain anonymous but your review may still be made public.

**Do you want your identity to be public for this peer review?**  For information about this choice, including consent withdrawal, please see our Privacy Policy

Reviewer #1: No

Reviewer #2: No

**********

[NOTE: If reviewer comments were submitted as an attachment file, they will be attached to this email and accessible via the submission site. Please log into your account, locate the manuscript record, and check for the action link "View Attachments". If this link does not appear, there are no attachment files.]

To ensure your figures meet our technical requirements, please review our figure guidelines: https://journals.plos.org/plosone/s/figures

You may also use PLOS’s free figure tool, NAAS, to help you prepare publication quality figures: https://journals.plos.org/plosone/s/figures#loc-tools-for-figure-preparation.

NAAS will assess whether your figures meet our technical requirements by comparing each figure against our figure specifications.

# Author comment: Author response to Decision Letter 1

**DOI:** 10.1371/journal.pone.0359269.r002

4 Jun 2026

Please find attached (Repsonse to reviewers) our detailed response to reviewers and the revised manuscript. All reviewer comments have been addressed.

Attachment Submitted filename: Response to reviewers.docx

# Peer review: Decision Letter 1

**DOI:** 10.1371/journal.pone.0359269.r003

**Authors:** Miriam Hickey

1 Jul 2026

Dear Dr. Culley,

Please now address comments from Reviewer #2

Please submit your revised manuscript by Aug 15 2026 11:59PM. If you will need more time than this to complete your revisions, please reply to this message or contact the journal office at plosone@plos.org. When you're ready to submit your revision, log on to https://www.editorialmanager.com/pone/ and select the 'Submissions Needing Revision' folder to locate your manuscript file.

- A letter that responds to each point raised by the academic editor and reviewer(s). You should upload this letter as a separate file labeled 'Response to Reviewers'.
- A marked-up copy of your manuscript that highlights changes made to the original version. You should upload this as a separate file labeled 'Revised Manuscript with Track Changes'.
- An unmarked version of your revised paper without tracked changes. You should upload this as a separate file labeled 'Manuscript'.

If you would like to make changes to your financial disclosure, please include your updated statement in your cover letter. Guidelines for resubmitting your figure files are available below the reviewer comments at the end of this letter.

If applicable, we recommend that you deposit your laboratory protocols in protocols.io to enhance the reproducibility of your results. Protocols.io assigns your protocol its own identifier (DOI) so that it can be cited independently in the future. For instructions see: https://journals.plos.org/plosone/s/submission-guidelines#loc-laboratory-protocols. Additionally, PLOS ONE offers an option for publishing peer-reviewed Lab Protocol articles, which describe protocols hosted on protocols.io. Read more information on sharing protocols at https://plos.org/protocols?utm_medium=editorial-email&utm_source=authorletters&utm_campaign=protocols.

As the corresponding author, your ORCID iD is verified in the submission system and will appear in the published article. PLOS supports the use of ORCID, and we encourage all coauthors to register for an ORCID iD and use it as well. Please encourage your coauthors to verify their ORCID iD within the submission system before final acceptance, as unverified ORCID iDs will not appear in the published article. *Only*  the individual author can complete the verification step; PLOS staff *cannot*  verify ORCID iDs on behalf of authors.

We look forward to receiving your revised manuscript.

Kind regards,

Miriam A. Hickey, PhD

Academic Editor

PLOS One

Journal Requirements:

If the reviewer comments include a recommendation to cite specific previously published works, please review and evaluate these publications to determine whether they are relevant and should be cited. There is no requirement to cite these works unless the editor has indicated otherwise.

Please review your reference list to ensure that it is complete and correct. If you have cited papers that have been retracted, please include the rationale for doing so in the manuscript text, or remove these references and replace them with relevant current references. Any changes to the reference list should be mentioned in the rebuttal letter that accompanies your revised manuscript. If you need to cite a retracted article, indicate the article’s retracted status in the References list and also include a citation and full reference for the retraction notice.

[Note: HTML markup is below. Please do not edit.]

Reviewers' comments:

Reviewer's Responses to Questions

**Comments to the Author**

Reviewer #2: (No Response)

**********

2. Is the manuscript technically sound, and do the data support the conclusions?

Reviewer #2: Yes

**********

3. Has the statistical analysis been performed appropriately and rigorously?

Reviewer #2: Yes

**********

4. Have the authors made all data underlying the findings in their manuscript fully available?

The PLOS Data policy

Reviewer #2: Yes

**********

5. Is the manuscript presented in an intelligible fashion and written in standard English?

Reviewer #2: Yes

**********

Reviewer #2: The revised version of the manuscript entitled “AZP2006 in Parkinson’s Disease Models: A Promising New Neuroprotective Treatment Involving Lysosomal Homeostasis and the Progranulin/Prosaposin Complex” by Callizot and colleagues had been substantially improved. A few comments are listed below:

1) Introduction: Page 2, end 2nd paragraph: please set “lysosomal-autophagic system” for “lysosomal-autophagy” system

2) Introduction: Page 2, first sentence of 3rd paragraph: please check grammar

3) Introduction: Page 4, first sentence: did you mean: ..therapy development…”

4) Figure legends with cellular analysis: what does n = 4-6 / group mean? I guess, 4-6 independent experiments were included. How many cells were included per independent experiment?

5) Page 16, last paragraph. Referencing of Fig 3 panels in the main text is not correct. Please re-check!

**********

what does this mean?). If published, this will include your full peer review and any attached files.

If you choose “no”, your identity will remain anonymous but your review may still be made public.

**Do you want your identity to be public for this peer review?**  For information about this choice, including consent withdrawal, please see our Privacy Policy

Reviewer #2: No

**********

[NOTE: If reviewer comments were submitted as an attachment file, they will be attached to this email and accessible via the submission site. Please log into your account, locate the manuscript record, and check for the action link "View Attachments". If this link does not appear, there are no attachment files.]

To ensure your figures meet our technical requirements, please review our figure guidelines: https://journals.plos.org/plosone/s/figures

You may also use PLOS’s free figure tool, NAAS, to help you prepare publication quality figures: https://journals.plos.org/plosone/s/figures#loc-tools-for-figure-preparation.

NAAS will assess whether your figures meet our technical requirements by comparing each figure against our figure specifications.

# Author comment: Author response to Decision Letter 2

**DOI:** 10.1371/journal.pone.0359269.r004

4 Aug 2026

Dear reviewer,

Than you for your review. The additional comments raised have been fully addressed. The modified manuscript has been resubmitted, along with the track changes version and we have submitted the "response to reviewers" letter to respond in detail to each point.

Than you for taking the time to read our manuscipt.

Yours sincerely,

Georgia Culley

Attachment Submitted filename: Response_to_reviewers_auresp_2.docx

# Peer review: Decision Letter 2

**DOI:** 10.1371/journal.pone.0359269.r005

**Authors:** Miriam Hickey

26 Aug 2026

Dear Dr. Culley,

Thank you for submitting your manuscript to PLOS One. After careful consideration, we feel that it has merit but does not fully meet PLOS One’s publication criteria as it currently stands. Therefore, we invite you to submit a revised version of the manuscript that addresses the points raised during the review process.

Please submit your revised manuscript by Oct 10 2026 11:59PM. If you will need more time than this to complete your revisions, please reply to this message or contact the journal office at plosone@plos.org. When you're ready to submit your revision, log on to https://www.editorialmanager.com/pone/ and select the 'Submissions Needing Revision' folder to locate your manuscript file.

- A letter that responds to each point raised by the academic editor and reviewer(s). You should upload this letter as a separate file labeled 'Response to Reviewers'.
- A marked-up copy of your manuscript that highlights changes made to the original version. You should upload this as a separate file labeled 'Revised Manuscript with Track Changes'.
- An unmarked version of your revised paper without tracked changes. You should upload this as a separate file labeled 'Manuscript'.

If you would like to make changes to your financial disclosure, please include your updated statement in your cover letter. Guidelines for resubmitting your figure files are available below the reviewer comments at the end of this letter.

If applicable, we recommend that you deposit your laboratory protocols in protocols.io to enhance the reproducibility of your results. Protocols.io assigns your protocol its own identifier (DOI) so that it can be cited independently in the future. For instructions see: https://journals.plos.org/plosone/s/submission-guidelines#loc-laboratory-protocols. Additionally, PLOS One offers an option for publishing peer-reviewed Lab Protocol articles, which describe protocols hosted on protocols.io. Read more information on sharing protocols at https://plos.org/protocols?utm_medium=editorial-email&utm_source=authorletters&utm_campaign=protocols.

As the corresponding author, your ORCID iD is verified in the submission system and will appear in the published article. PLOS supports the use of ORCID, and we encourage all coauthors to register for an ORCID iD and use it as well. Please encourage your coauthors to verify their ORCID iD within the submission system before final acceptance, as unverified ORCID iDs will not appear in the published article. *Only*  the individual author can complete the verification step; PLOS staff *cannot*  verify ORCID iDs on behalf of authors.

We look forward to receiving your revised manuscript.

Kind regards,

Miriam A. Hickey, PhD

Academic Editor

PLOS One

Journal Requirements:

1. If the reviewer comments include a recommendation to cite specific previously published works, please review and evaluate these publications to determine whether they are relevant and should be cited. There is no requirement to cite these works unless the editor has indicated otherwise.

2. Please review your reference list to ensure that it is complete and correct. If you have cited papers that have been retracted, please include the rationale for doing so in the manuscript text, or remove these references and replace them with relevant current references. Any changes to the reference list should be mentioned in the rebuttal letter that accompanies your revised manuscript. If you need to cite a retracted article, indicate the article’s retracted status in the References list and also include a citation and full reference for the retraction notice.

[Note: HTML markup is below. Please do not edit.]

Reviewers' comments:

Reviewer's Responses to Questions

**Comments to the Author**

Reviewer #2: All comments have been addressed

**********

2. Is the manuscript technically sound, and do the data support the conclusions?

Reviewer #2: Yes

**********

3. Has the statistical analysis been performed appropriately and rigorously?

Reviewer #2: Yes

**********

4. Have the authors made all data underlying the findings in their manuscript fully available?

The PLOS Data policy

Reviewer #2: Yes

**********

5. Is the manuscript presented in an intelligible fashion and written in standard English?

Reviewer #2: Yes

**********

Reviewer #2: The revised manuscript by Callizot and co-workers has been significantly improved and all commwnts were adressed. Please correct the references for Fig 3 in the main text (e.g. p16). Fig 3 does not show panels F and G.

**********

what does this mean?). If published, this will include your full peer review and any attached files.

If you choose “no”, your identity will remain anonymous but your review may still be made public.

**Do you want your identity to be public for this peer review?**  For information about this choice, including consent withdrawal, please see our Privacy Policy

Reviewer #2: No

**********

[NOTE: If reviewer comments were submitted as an attachment file, they will be attached to this email and accessible via the submission site. Please log into your account, locate the manuscript record, and check for the action link "View Attachments". If this link does not appear, there are no attachment files.]

To ensure your figures meet our technical requirements, please review our figure guidelines: https://journals.plos.org/plosone/s/figures

You may also use PLOS’s free figure tool, NAAS, to help you prepare publication quality figures: https://journals.plos.org/plosone/s/figures#loc-tools-for-figure-preparation.

NAAS will assess whether your figures meet our technical requirements by comparing each figure against our figure specifications.

# Author comment: Author response to Decision Letter 3

**DOI:** 10.1371/journal.pone.0359269.r006

10 Sep 2026

We thank you for this positive assessment of our manuscript.

We carefully reviewed all references to Figure 3 in the manuscript. The incorrect references to Figure 3 panels had already been corrected during the previous revision round. Nevertheless, to ensure complete clarity and address the reviewer's concern, we performed an additional review of all Figure 3 citations and added an explicit reference to Figure 3B (representative images) in the relevant section of the text.

We hope this clarification fully addresses your comment.

Attachment Submitted filename: Response_to_reviewers_auresp_3.docx

# Editor report: Decision Letter 3

**DOI:** 10.1371/journal.pone.0359269.r007

**Authors:** Miriam Hickey

13 Sep 2026

AZP2006 in Parkinson’s Disease Models: A Promising New Neuroprotective Treatment Involving Lysosomal Homeostasis and the Progranulin/Prosaposin Complex

PONE-D-25-44136R3

Dear Dr. Culley,

We’re pleased to inform you that your manuscript has been judged scientifically suitable for publication and will be formally accepted for publication once it meets all outstanding technical requirements.

Within one week, you’ll receive an e-mail detailing the required amendments. When these have been addressed, you’ll receive a formal acceptance letter and your manuscript will be scheduled for publication.

An invoice will be generated when your article is formally accepted. Please note, if your institution has a publishing partnership with PLOS and your article meets the relevant criteria, all or part of your publication costs will be covered. Please make sure your user information is up-to-date by logging into Editorial Manager at Editorial Manager® and clicking the ‘Update My Information' link at the top of the page. For questions related to billing, please contact billing support.

If your institution or institutions have a press office, please notify them about your upcoming paper to help maximize its impact. If they’ll be preparing press materials, please inform our press team as soon as possible -- no later than 48 hours after receiving the formal acceptance. Your manuscript will remain under strict press embargo until 2 pm Eastern Time on the date of publication. For more information, please contact onepress@plos.org.

Kind regards,

Miriam A. Hickey, PhD

Academic Editor

PLOS One

Additional Editor Comments (optional):

All comments are now satisfactorily addressed.

Reviewers' comments:

# Editor report: Acceptance letter

**DOI:** 10.1371/journal.pone.0359269.r008

**Authors:** Miriam Hickey

PONE-D-25-44136R3

PLOS One

Dear Dr. Culley,

I'm pleased to inform you that your manuscript has been deemed suitable for publication in PLOS One. Congratulations! Your manuscript is now being handed over to our production team.

At this stage, our production department will prepare your paper for publication. This includes ensuring the following:

* All references, tables, and figures are properly cited

* All relevant supporting information is included in the manuscript submission,

* There are no issues that prevent the paper from being properly typeset

You will receive further instructions from the production team, including instructions on how to review your proof when it is ready. Please keep in mind that we are working through a large volume of accepted articles, so please give us a few days to review your paper and let you know the next and final steps.

Lastly, if your institution or institutions have a press office, please let them know about your upcoming paper now to help maximize its impact. If they'll be preparing press materials, please inform our press team within the next 48 hours. Your manuscript will remain under strict press embargo until 2 pm Eastern Time on the date of publication. For more information, please contact onepress@plos.org.

You will receive an invoice from PLOS for your publication fee after your manuscript has reached the completed accept phase. If you receive an email requesting payment before acceptance or for any other service, this may be a phishing scheme. Learn how to identify phishing emails and protect your accounts at https://explore.plos.org/phishing.

If we can help with anything else, please email us at customercare@plos.org.

Thank you for submitting your work to PLOS One and supporting open access.

Kind regards,

PLOS One Editorial Office Staff

on behalf of

Dr. Miriam A. Hickey

Academic Editor

PLOS One
